# Mutations in a P-Type ATPase Gene Cause Axonal Degeneration

**DOI:** 10.1371/journal.pgen.1002853

**Published:** 2012-08-09

**Authors:** Xianjun Zhu, Richard T. Libby, Wilhelmine N. de Vries, Richard S. Smith, Dana L. Wright, Roderick T. Bronson, Kevin L. Seburn, Simon W. M. John

**Affiliations:** 1 The Jackson Laboratory, Bar Harbor, Maine, United States of America; 2 The Howard Hughes Medical Institute, Bar Harbor, Maine, United States of America; 3 Department of Ophthalmology, Tufts University of Medicine, Boston, Massachusetts, United States of America; University of Michigan, United States of America

## Abstract

Neuronal loss and axonal degeneration are important pathological features of many neurodegenerative diseases. The molecular mechanisms underlying the majority of axonal degeneration conditions remain unknown. To better understand axonal degeneration, we studied a mouse mutant wabbler-lethal (*wl*). Wabbler-lethal (*wl*) mutant mice develop progressive ataxia with pronounced neurodegeneration in the central and peripheral nervous system. Previous studies have led to a debate as to whether myelinopathy or axonopathy is the primary cause of neurodegeneration observed in *wl* mice. Here we provide clear evidence that wabbler-lethal mutants develop an axonopathy, and that this axonopathy is modulated by *Wld^s^* and *Bax* mutations. In addition, we have identified the gene harboring the disease-causing mutations as *Atp8a2*. We studied three *wl* alleles and found that all result from mutations in the *Atp8a2* gene. Our analysis shows that ATP8A2 possesses phosphatidylserine translocase activity and is involved in localization of phosphatidylserine to the inner leaflet of the plasma membrane. *Atp8a2* is widely expressed in the brain, spinal cord, and retina. We assessed two of the mutant alleles of *Atp8a2* and found they are both nonfunctional for the phosphatidylserine translocase activity. Thus, our data demonstrate for the first time that mutation of a mammalian phosphatidylserine translocase causes axon degeneration and neurodegenerative disease.

## Author Summary

Axonal degeneration is an important pathological feature of many neurodegenerative diseases, such as Alzheimer disease, Parkinson's disease, and amyotrophic lateral sclerosis. In most of these disease conditions, molecular mechanisms of axonal degeneration remain largely unknown. Spontaneous mouse mutants are important in human disease studies. Identification of a disease-causing gene in mice can lead to the identification of the human ortholog as the disease gene in humans. This approach has the power to identify unexpected genes and pathways involved in disease. Our study centered on wabbler lethal (*wl*) mutant mice, which display axonal degeneration in both the central and peripheral nervous systems. We identified the disease-causing gene in mice with different *wl* mutations. The mutations are in *Atp8a2*, a gene encoding a phosphatidylserine translocase. This protein functions to keep phosphatidylserine enriched to the inner leaflet of the plasma membrane. Our study demonstrates a new role for phospholipid asymmetry in maintaining axon health, and it also reveals a novel function for phosphatidyleserine translocase in neurodegenerative diseases.

## Introduction

Neuronal loss and axonal degeneration are important pathological features of neurodegenerative diseases, such as Alzheimer disease, Parkinson's disease, amyotrophic lateral sclerosis and glaucoma. Axonopathies, conditions in which axon injury occurs first during disease progression, have been extensively studied, but the mechanism(s) underlying axon degeneration remain to be elucidated in most of these conditions. In order to rationally develop effective therapeutics for these conditions, it is critical to determine the molecular mechanisms underlying axonopathies.

Spontaneous mouse mutants have long been used to gain insight into human disease. Spontaneous mutants provide valuable platforms for identifying disease-associated pathways. Identifying the gene(s) that cause a certain phenotype in mice can lead to a greater understanding of the pathophysiology of a disease. Identification of a disease causing mutation in mice often precedes the identification of the orthologous gene as a cause of a corresponding disease in humans [1]–[10]. As with all forward genetic tools, the power of analyzing mutants is that it allows for the identification of pathways involved in disease that may not have been identified through experiments based on current knowledge. To understand axon pathophysiology better, we studied wabbler-lethal (*wl*) mutant mice that display axon degeneration. The autosomal recessive *wl* mutation arose spontaneously in a mouse colony at The Jackson Laboratory in 1952 [11]. Homozygous *wl* mice are characterized by severe neurological abnormalities that include ataxia and body tremors. Abnormalities are first apparent around twelve days of age and mutant mice generally die around 4 weeks of age [11]–[13]. Histopathology of the *wl* nervous system is consistent with *wl* being an axonopathy [12], [14], though it has also been suggested to be primarily a myelinopathy [11], [15]. The genetic defect that causes *wl* has been unknown.

Here, we performed an extensive analysis of wabbler lethal mice and show that they develop a progressive axonal degeneration in several different areas of the nervous system. The presence of prominent axon degeneration, without initial myelin damage, and the absence of obvious cell death point to an axonopathy. To gain further understanding of the molecular pathways that can underlie the axonopathy in *wl* mice, the autosomal recessive *wl* mutation was positionally cloned. Using a combination of genetic and biochemical approaches, we demonstrated that the pathological lesion of this mutation is due to loss of function mutations in the gene encoding the murine phosphatidylserine translocase (flippase) *Atp8a2*. Loss of phosphatidylserine flippase activity leads to decreased axonal transport as indicated by the accumulation of phosphorylated neurofilament in motor neurons and retinal ganglion cell bodies. These data establish a novel role for a phosphatidylserine flippase in maintaining axonal health in both the central and peripheral nervous systems.

## Results

### The wabbler-lethal (*wl*) phenotype is an axonopathy

**Figure 1:**
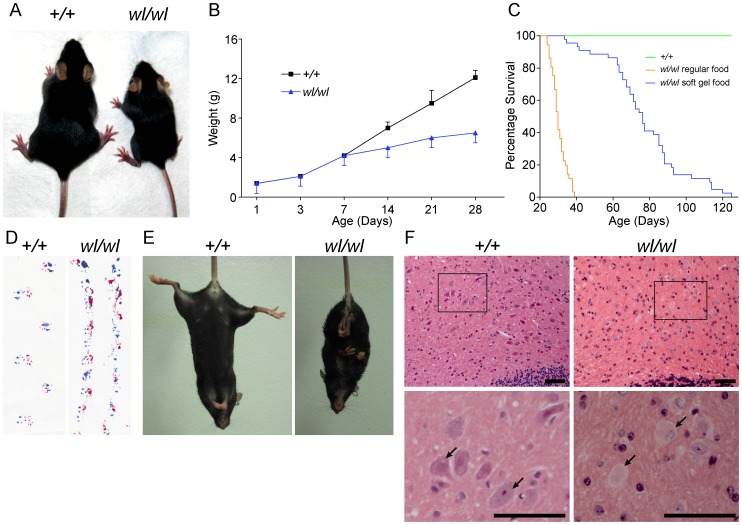
Phenotypic characterization of *wl/wl* mice on a C57BL/6J background. (A) *wl/wl* mice are noticeably smaller at twenty one days after birth (P21) than their wild type (*+/+*) litter mates. (B) *wl/wl* mice fall behind their wild type (*+/+*) litter mates in weight gain from seven days after birth (P7) onward, and are clearly distinguishable from wild type at about 12 days of age (n = 13 for *+/+*; n = 9 for *wl/wl*). For animals at P7 or older, we used male animals. For younger animals, we used both males and females. The weight gain is consistent with previous reports of the *wl* mutation on other genetic backgrounds [77]. (C) Compared to littermate controls (green line), mutant mice (orange line) had a much lower survival rate. Even with the addition of a soft maintenance diet, 20% of *wl/wl* mutants (blue line) die by 65 days and all *wl/wl* mutants die by 130 days of age (n = 46 for *+/+*; n = 44 for *wl/wl*). (D; E) Phenotypic consequences of the *wl* mutation: *wl/wl* mice drag their hind feet when walking as evident by ink traces (D: blue, front paws; red, rear paws), and (E) show clasping of the hind limbs when picked up by the tail, characteristic of a neurological deficit. (F) Hematoxylin and eosin staining of deep cerebellar nuclei in wild type (*+/+*) and *wl/wl* mice of two months of age show central chromatolysis in *wl/wl* mice (pale neurons) but not in wild type mice. Arrows indicate healthy neurons in *+/+* mice and abnormal neurons in *wl/wl* mice. Black boxes in the top panels indicate areas that have been magnified in the bottom panels. Scale bar is 50 µm.

Mice homozygous for the *wl* mutation (*wl/wl*; *wl* mutants) grow much slower than their littermate controls and are first phenotypically recognizable at about 12 days of age, due to their smaller body size (Figure 1A and B). Supplementation of dry food with a soft moist diet that was placed on the cage floor to allow easy access, allowed homozygous mutants to survive past the previously reported 30 days (Figure 1C) [11]. Even on this diet, however, twenty percent of *wl/wl* mice died by 65 days of age, and all died by 130 days. Homozygous mutant mice develop a body tremor, an abnormal gait (Figure 1D), and display an abnormal hind limb-clasping reflex indicative of a neurological deficit that is very obvious at two months of age (Figure 1E).

Central chromatolysis is regarded as a characteristic feature of axonopathies [16]. Here we documented chromatolysis in the lateral cerebellar nucleus (Figure 1F), medial cerebellar nucleus and lateral vestibular nucleus (Figure S1) in *wl* mutants but not controls. Affected neurons have pale staining and acentric nuclei in hematoxylin and eosin stained sections (Figure 1F, arrows). Importantly, despite cell bodies with obvious chromatolysis in the lateral cerebellar nucleus, intermediate nucleus, spinal cord and other regions of the cerebellum, no obvious cell loss was noted in any of these regions, and cleaved caspase 3 staining did not detect an increased number of apoptotic cells (Figure S2). Dystrophic axons were evident in the corticospinal tract, spinalcerebellar tract (Figure S3) and spinal white matter (Figure S4). These data are consistent with a primary axonopathy without cell loss.

**Figure 2:**
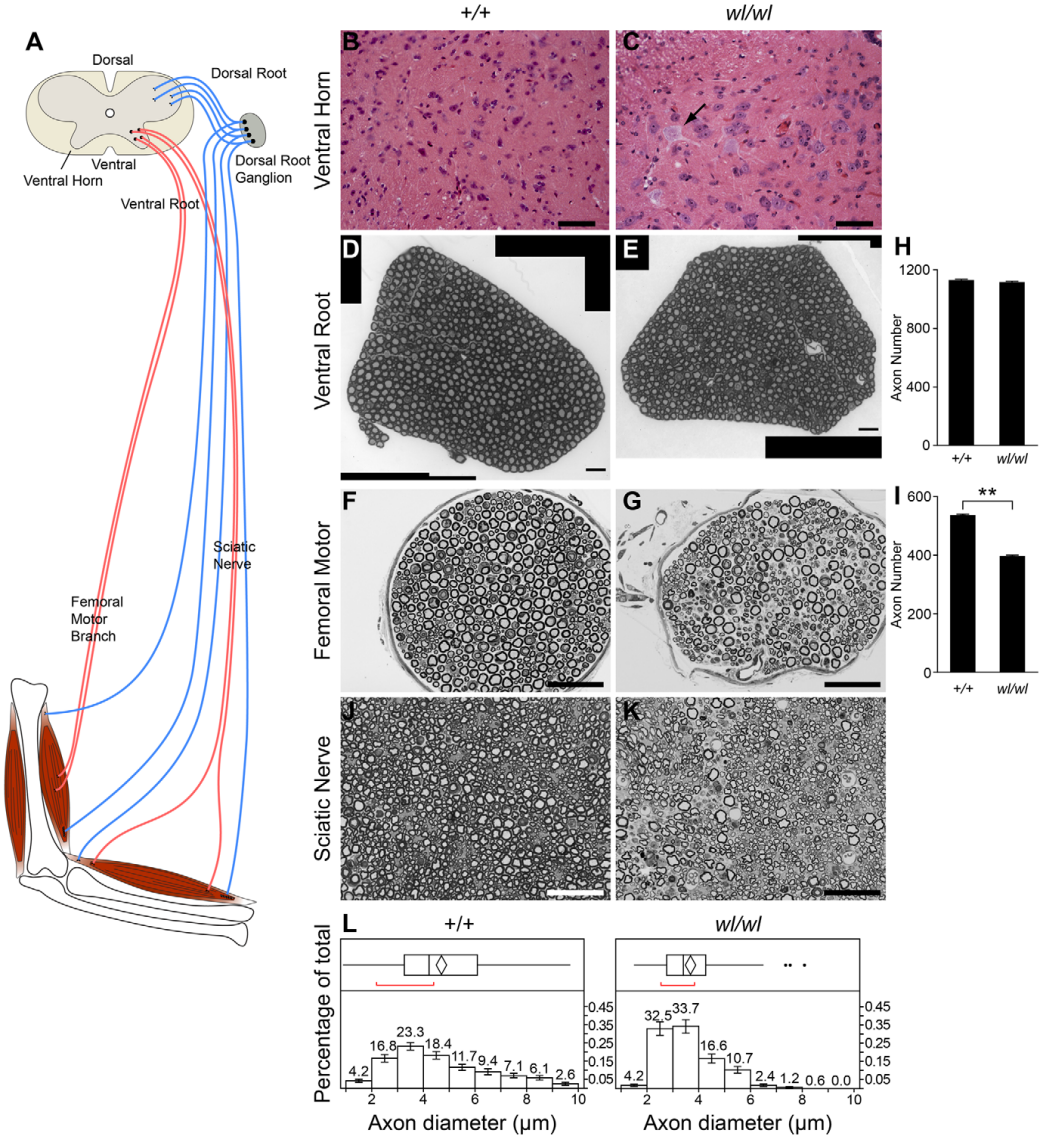
Characteristics of axonopathy in *wl/wl* mice. (A) Diagram showing the structures of the spinal cord and associated peripheral nerves. (B,C) H&E staining of lumbar spinal cord sections. Note the central chromotalysis in the *wl/wl* mutant motor neurons (arrow). (D, E) Semi-thin (1 µm) sections of the L4 ventral root stained with toluidine blue. No morphological differences between wild type and mutant nerves are apparent. This is reflected in the similar number of axons present in the wild type and mutant nerves (H; n = 4 for each genotype). (F, G) Semi-thin (1 µm) sections of the femoral nerve motor branch stained with toluidine blue clearly showed a morphological difference between wild type and mutant nerves, with a reduced number of axons in the *wl/wl* mice. The average axon number in the motor branch was significantly decreased in *wl/wl* mice compared to wild type mice (p<0.01; n = 4 for both genotypes). (J, K) Semi-thin (1 µm) sections of the sciatic nerve stained with toluidine blue in 60 day old wild type and *wl/wl* mutant mice. (L) Quantification of the mean axonal diameter of these axons shows that *wl/wl* mice have far fewer large diameter axons compared to wild type mice. Average axon diameter was significantly decreased in *wl/wl* mice compared to controls (**, p<0.01). The axon diameter for over 400 axons from wild type and *wl/wl* mice were analyzed by TEM. Scale bar, 50 µm.

Central chromatolysis was also observed in the spinal ventral horn at different spinal levels (Figure 2B and C), again without obvious cell loss. Spinal motor neurons are located in the ventral horns and their axons project into the ventral root and then the spinal nerves. Supporting an absence of cell loss, even at 3 months of age when the disease is very severe (see below), axon counts for the ventral root (close to the cell bodies) were indistinguishable between *wl* mutant (count 1090±14, n = 4) and control mice (count 1101±7, n = 4, P = 0.22).

### The wabbler-lethal phenotype is a distal axonopathy

To determine if axon injury was first visible closer (proximal) to the cell body, or farther away (distal) from the cell body, we analyzed axons in the proximal ventral root and the distal femoral motor branch, which primarily consists of long motor axons. At two months of age, no obvious axon loss or morphological changes were present in the L4 ventral root (Figure 2D, E and H). However, the same *wl/wl* mice had lost 20% of axons in their distal femoral motor branch (Figure 2F, G, and I) with many axons having an irregular shape and having darkly stained axoplasm (Figure 2G). The femoral motor branch in 8-day old mutant and control mice were similar in both the total axon number and axon morphology, indicating the axon damage is not developmental and occurs with aging (Figure S5). The axonal degeneration in the femoral nerve is thus initially prominent in the distal part of the nerve with no apparent loss of axons in the ventral root. These data suggest that distal axonal degeneration is the main cause of the disease in *wl* mice.

Analysis of the distal sciatic nerve of *wl* mice at two months of age revealed that large diameter axons were preferentially lost (Figure 2J–L; Figure S6C). In general, large diameter motor neurons are known to have thicker myelin than smaller diameter motor neurons. Consistent with loss of large axons, myelin thickness is reduced in two-month old mutant mice from a mean value of 0.99±0.02 µm to a mean value of 0.57±0.01 µm (p<0.01). Despite severe neurological abnormalities at two months of age, no obvious demyelination defects were observed by assessing the ratio of inner axon diameter (inside myelin) to the total axon diameter (G-ratio, not shown). Additionally the condition of myelin in *wl* mutants and controls was indistinguishable by transmission electron microscopy at 2 month of age (Figure S6). These data suggest that demyelination is not the primary cause of the observed phenotype.

### Wabbler-lethal mutants have disrupted axon transport of neurofilaments

**Figure 3:**
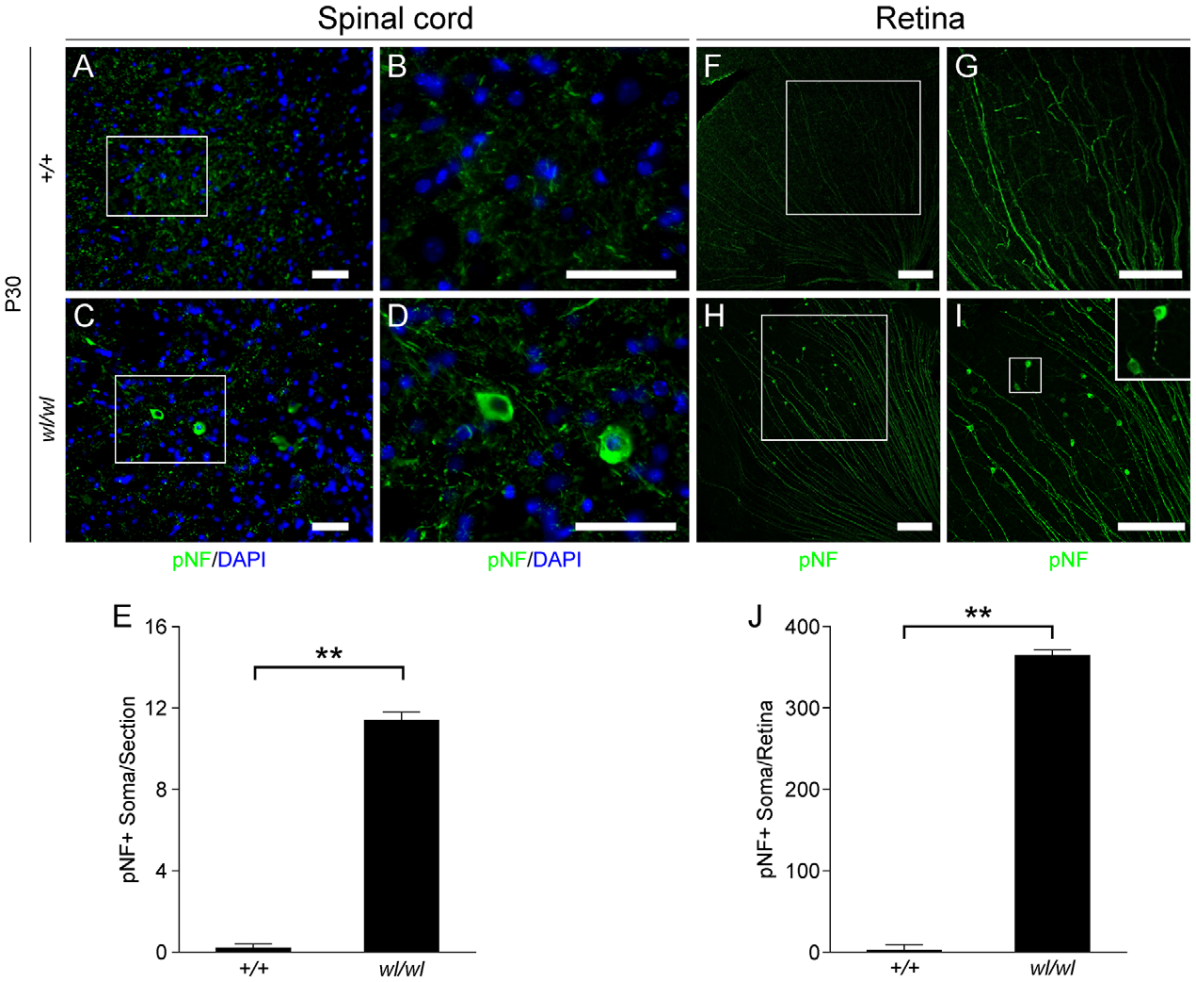
Phosphorylated neurofilament (pNF) accumulates in somas of spinal cord neurons and retinal ganglion cells in *wl/wl* mice. Note the white boxes in A, C, F and H define areas that have been magnified in B, D, G and H, respectively. (A, B) At P30 pNF localized to only axons of lumbar motor neurons in wild type mice, but was not found in the somas of neurons. (C, D) At P30 in *wl/wl* mutant animals pNF was present in the axons of motor neurons, but also accumulated in the somas of some motor neurons. (E) Lumbar motor neuron sections stained with pNF antibody were used to count the number of soma in which pNF accumulated. There was a significant increase in the number of somas accumulating pNF in the motor neurons of *wl/wl* mice (12±0.4) compared to wild type mice (0.2±0.2). (F, G) In P30 wild type mice pNF labeled only ganglion cell axons in the retina, but in *wl/wl* mutants (H, I) pNF was also present in a subset of ganglion cell somas, especially in the peripheral region of the retina. In addition, focal swelling was observed in some axons. An enlargement of a focal swelling is shown in the upper right hand corner of I, which corresponds to the box in I. (J) Quantification of pNF+ somas confirms a massive increase in the number of somas accumulating pNF in *wl/wl* mice (364±7) compared to controls (2±0.5). **, P<0.01; Scale bar, 50 µm.

The distal degeneration of motor axons in the *wl* mutants prompted us to examine phosphorylated neurofilament (pNF) as a marker of axonal transport in spinal motor neurons. The localization of intermediate to high molecular weight pNF has been widely used to assess axonal transport [17], [18]. Under normal conditions, pNF is rarely detected in the cell body as it is normally efficiently transported from the soma to the more distal axon. When axonal transport is disrupted, pNF accumulates in the cell body [17], [18]. Staining of lumbar motor neurons of one-month-old wild type mice with pNF antibody detected neurofilament in axons (Figure 3A and B), while accumulation in the somas was very rare (Figure 3E). In contrast, pNF was present in both the axons and somas of lumbar motor neurons in *wl* mutant mice (3C, D and E; 12±0.4 pNF positive soma in *wl/wl* mice; 0.2±0.2 pNF positive soma in +/+ control mice, p<0.01). Both motor neurons in the ventral horn and neurons in the dorsal gray column display accumulation of pNF in their cell bodies. In addition, pNF accumulation was observed in different regions of the brain, such as the medial cerebellar nucleus, intermediate reticular nucleus and raphe magnus nucleus (Figure S7), indicating a common defect in axonal transport of neurofilament.

The axons of retinal ganglion cells (RGCs) also degenerate in the optic nerves of *wl* mice [12]. By one month, RGCs from *wl* mutants showed disrupted axon transport as evidenced by pNF accumulation in their somas. This accumulation occurs mainly in the peripheral retina (Figure 3H, I and J; 364±7 pNF positive soma in *wl/wl* mice; 2±0.5 pNF positive soma in +/+ control mice, p<0.01). To determine if axon injury, evident as axon transport defects, occurs before or after morphological changes, the optic nerves of *wl* mutants were assessed for axon damage using a histochemical stain paraphenylenediamine (PPD). PPD stains all myelin sheaths but is very sensitive for detecting axon injury (Figure S8), as the axoplasm of injured axons stains darkly [19]–[23]. At 30 days of age, the optic nerves of *wl* mice are indistinguishable from those in wild type mice. Despite this normal appearance of their axons, the RGCs have axon transport defects as indicated by accumulation of pNF in their somas (Figure 3H–J). Together, the axon transport defects in neurons with structurally normal axons and myelin sheaths, the central chromatolysis and the distal axonal degeneration in the femoral and sciatic nerves are consistent with an axonopathy.

### *Wld^s^* and *Bax* delay the axonopathy in *wl* mice

**Figure 4:**
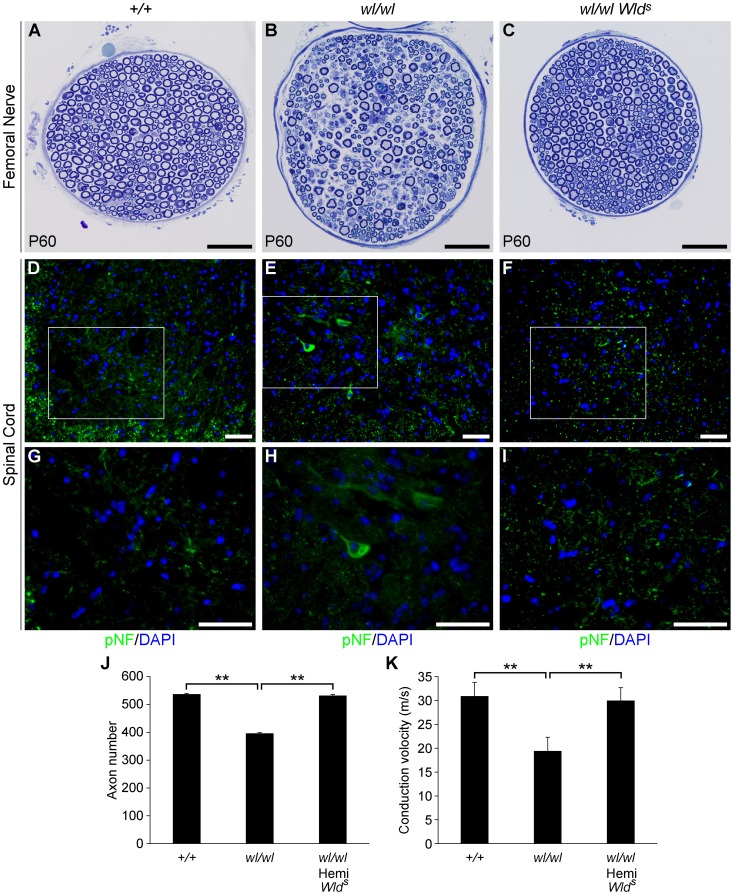
The *Wld^s^* mutation delays axonal degeneration in *wl* (*wl/wl*) mice. (A–C) Femoral nerves of wild type (*+/+*), *wl/wl* and *wl/wl Wld^s^* (*wl* mice hemizygous for a *Wld^s^* allele) were examined for axonal degeneration at two months of age. Severe axonal degeneration occurred in *wl* mutant mice (B), while the *Wld^s^* allele significantly delays axonal degeneration (C). Axon morphology *wl Wld^s^* was similar to that in wild type control mice. (D–I) *Wld^s^* prevented accumulation of pNF (green) in spinal cord soma of *wl* mutant mice (note, white box in D–F indicates area magnified is G–I). Abnormal accumulation of pNF was observed in the soma of *wl* mice (E, H) but not *wl Wld^s^* mice (F, I). (J) Total number of myelinated axons in the femoral nerve of mice of each genotype at two months of age. Hemizygosity (hemi) for *Wld^s^* mice prevented axon loss by this age (n = 4 for each genotype assessed). Number of axons ± sem for mice of each genotype were 537±3 (*+/+*); 396±3.4 (*wl/wl*) and 532±4.4 (*wl/wl Wld^s^*). (K) Nerve conduction velocity (NCV) was also rescued by *Wld^s^*. In *wl* mice, increased distal latency indicated a reduction in nerve conduction velocity. In the presence of *Wld^s^*, NCV was restored to almost control levels (n = 4, for control mice; n = 5 for *wl/wl* mutant; n = 6 for *wl/wl Wld^s^* animals). **, P<0.01; Scale bar, 50 µm.

The Wallerian degeneration slow (*Wld^s^*) allele dominantly delays axonal degeneration after direct axonal trauma and in axonopathies [24]–[30]. To further examine the role of axonal injury in the *wl* mutant, the effect of the *Wld^s^* allele was tested by crossing the *Wld^s^* allele to *wl* mice. The *Wld^s^* mutation has a strong protective effect on the survival of femoral nerve axons (Figure 4A–C). At two months, *wl* mutant nerves displayed severe axon degeneration (Figure 4B and J). In comparison, the number of axons in *wl Wld^s^* nerves were nearly identical to the number of axons in controls (Figure 4C and J). To determine the effect of *Wld^s^* on early changes in motor neurons, we looked at pNF accumulation in lumbar spinal cord sections. *Wld^s^* prevented axon transport defects as assessed by pNF accumulation in *wl* mutants (for example, in the lumbar spinal cord; Figure 4D–I). This delay of axonopathy because of *Wld^s^* further argues that the *wl* phenotype is an axonopathy.

As a further functional evaluation of the protective effects of *Wld^s^* against spinal axonopathy, we assessed nerve conduction velocity (NCV) in the sciatic nerve. At two months of age NCV was decreased by 40% in the *wl* mice, falling from 30.7±3.1 m/s in control animals to 18.4±1.2 m/s (p<0.01) in *wl* mice. In contrast, *wl Wld^s^* mice retained normal conduction velocity (Figure 4K). These data indicate that *Wld^s^* delayed axon damage in *wl* mutants. However, at no point could *wl Wld^s^* mice be grossly distinguished from *wl* mice. Despite apparent healthy axon morphology, *wl Wld^s^* mice still displayed body tremor when walking. Their disease onset was still around 14 days after birth, they were smaller than wildtype controls, their locomotory performance did not improve, and they performed poorly in the wire hang test (Figure S9). In the latter, *wl* and *wl Wld^s^* mice were able to grip the cage top for an average of 12.2±3.4 and 13.2±2.9 seconds respectively (P>0.05), while wild type controls gripped for an average of 55.2±3 seconds (P = 0.01) compared to *wl* or *wl Wld^s^* mutants.

**Figure 5:**
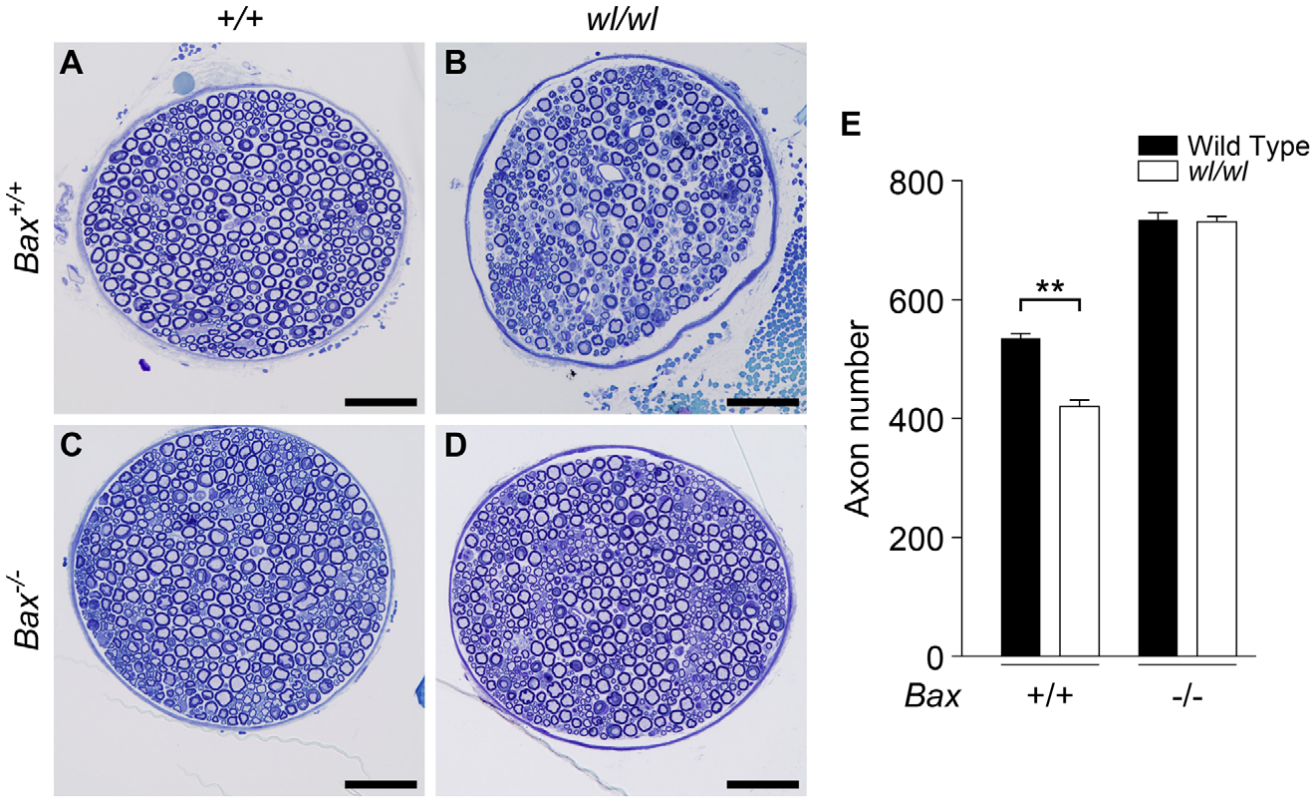
Genetic ablation of *Bax* protects axons from degeneration in *wl* mice. (A–D) Femoral nerves of mice of the indicated genotypes were examined at two months of age (genotypes above panels are for *Atp8a2*). The severe axonopathy in *wl* mutant mice (B), was significantly delayed by the *Bax* mutation (D). (E) Quantification of axon number in nerves from *wl* mutants either wild type or null for *Bax* (*Bax^+/+^* and *Bax^−/−^* respectively) showed that *Bax* deficiency prevented the axon loss in *wl/wl* mutants. *wl* mice had significantly fewer axons than wild type control animals (420±12 axons versus 535±8). In contrast, *wl* mice that are *Bax* deficient do not have any axon loss compared to *Bax* deficient mice (note *Bax* deficient mice have more neurons because of lack of normal developmental neuronal death; *Bax*−/− 734±12 axons, *wl/wl Bax* ^−/−^ 731±9). Four mice of each genotype were examined. **, P = 0.01. Scale bar is 50 µm.

The *Bax* gene is best known for its role in somal apoptosis, but also has an independent and intra-axonal role in axon degeneration [31], [32]. Genetic ablation of *Bax* strikingly protects axons in the femoral motor branch in two-month old *wl Bax^−/−^* mutants. At this age, *wl* mutants had severe axon loss while *wl Bax^−/−^* mice had no axon loss (Figure 5C, D, E). As for *Wld^s^*, the *Bax* mutation had no effect on lifespan or the gross neurological phenotype.

### The mutation causing the *wl* phenotype is in *Atp8a2*

**Figure 6:**
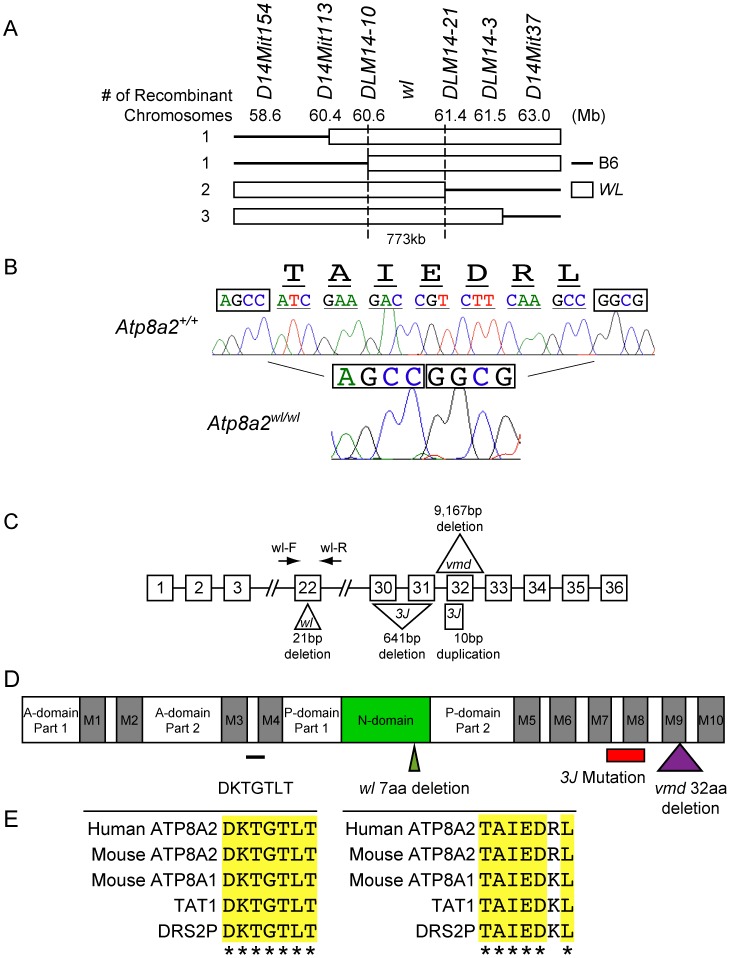
Positional cloning of the *wl* mutation. (A) Genotyping 688 affected F2 mice (1354 meiotic events) allowed the *wl* locus to be mapped to a 773 kb region on mouse chromosome 14 between DLM14-10 (60.6 Mb) and DLM14-21 (61.4 Mb). This region contains 10 genes. Solid lines represent parts of the chromosome containing B6 sequences, while open rectangles symbolize parts of the chromosome containing *wl* sequences in different mice. Numbers on the left side refer to the number of independent recombinant chromosomes obtained. (B) Sequence analysis of genomic DNA from mutant (*wl/wl*) and wild type (*+/+*) mice revealed a 21 bp deletion (ATCGAAGACCGTCTTCAAGCC) in exon 22 of *Atp8a2*, while the rest of the coding sequence is still in frame. Nucleotide bases immediately flanking the deletion region are boxed for easy comparison. The amino acids (TAIEDRL) encoded by the deleted base pairs are given at the top of the sequences. (C) Diagram showing the position and nature of the *wl* mutation and two additional *wl* alleles, *wl^vmd^* (*vmd*) and *wl^3J^* (*3J*). *vmd* genomic DNA harbors a 9,167 bp deletion, resulting in loss of exon 32 from the RNA transcript (Figure S10). *3J* has a 641 bp deletion starting from tenth base pair of exon 30. *3J* also contains a duplication of 10 bp (TCTTTGGTGT) in exon 32 (Figure S11). (D) Protein domains of ATP8A2 with the location of the three *wl* allele mutations indicated. ATP8A2 contains 10 putative transmembrane domains (M1–M10), an actuator domain (A-domain), a nucleotide-binding domain (N-domain), and a phosphorylation domain (P-domain). The position of *wl* mutation resulting in the deletion of seven highly conserved amino acids (TAIEDRL) in the N-domain is indicated by a green triangle; the position of the *vmd* mutation resulting in the deletion of 32 amino acids spanning the ninth transmembrane domain is indicated by a purple triangle, while the area of the protein affected by the *3J* deletion is indicated by a red bar (the small 3J duplication overlaps the vmd mutation). The location of seven highly conserved amino acids (DKTGTLT) is indicated with a black line. (E) Conservation of the seven amino acid DKTGTLT motif, which is highly conserved among all ATPases, as well as the seven amino acid sequence (TAIEDRL) deleted in *wl*, between mouse, human, *C. elegans* (TAT1) and yeast (DSR2P).

Previous linkage analysis localized *wl* to chromosome 14 [33]. Analysis of 688 affected *wl* F2 mice narrowed the interval where the mutation resides to a 773 kb region on chromosome 14 between DLM14-10 (60.6 Mb) and DLM14-21 (61.4 Mb) (Figure 6A). This region is known to contain 10 protein-coding genes. The coding regions and splice sites of each of these genes were sequenced and a 21 bp deletion in exon 22 of *Atp8a2* (Figure 6B and C) was identified. No mutations were found in the other 9 genes. This genomic deletion in *Atp8a2* leads to the elimination of seven highly conserved amino acids (TAIEDRL) from the nucleotide-binding domain (N-domain) of ATP8A2 (Figure 6D, E). Two additional alleles of *wl* were available: *wl^vmd^* (*vmd*) and *wl^3J^* (*3J*). We found that the *wl^vmd^* mutation is a large 9167 bp genomic deletion that results in removal of the entire exon 32 of *Atp8a2* (Figure 6C and Figure S10). This results in a 32 amino acid deletion in the ninth transmembrane domain of ATP8A2 (Figure 6D). *wl^3J^* mice were found to have a 641 bp deletion starting at the tenth base pair of exon 30 of *Atp8a2*, leading to the deletion of part of exon 30 and the whole exon 31. Furthermore, *wl^3J^* mice had a 10 bp duplication in exon 32 (Figure 6C and Figure S11). Genetic mapping and sequence analysis of these three alleles of *wl* clearly show that mutations in *Atp8a2* cause the *wl* phenotype.

### ATP8A2 is localized to the membrane

**Figure 7:**
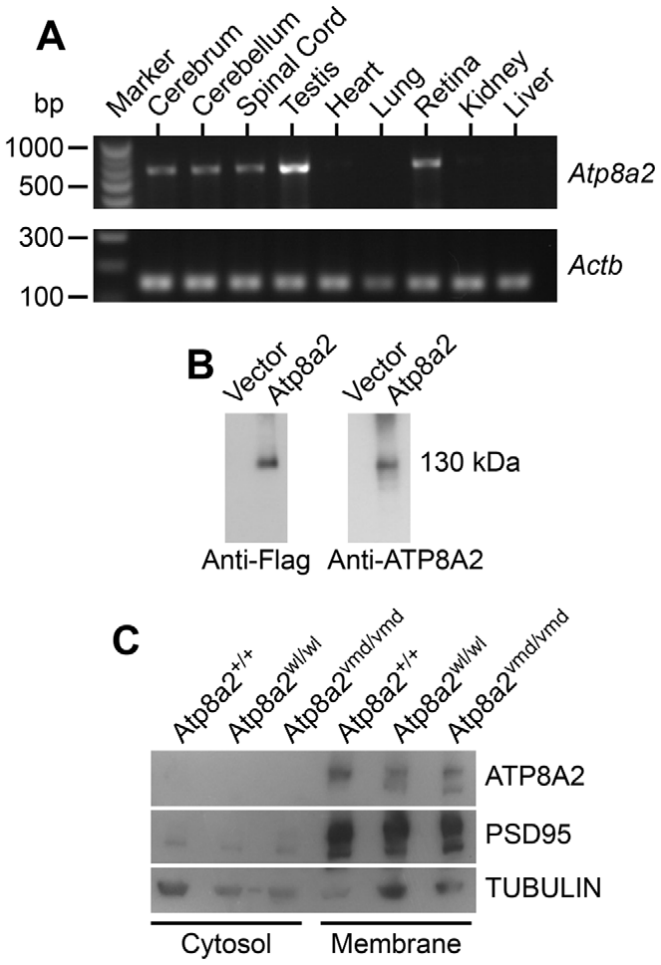
ATP8A2 expression and localization. (A) Reverse transcript (RT)-PCR analysis showed that *Atp8a2* is expressed in the cerebrum, cerebellum, spinal cord, testis and retina (Upper panel). The control for RT-PCR efficiency was β-actin (*Actb*; lower panel). (B) HEK293T cells were transfected either with mouse *Atp8a2* cloned into pCMV6-AN-DDK vector, or empty vector in order to characterize the newly generated anti-ATP8A2 polyclonal rabbit antibody. Total protein (10 µg) isolated from these cells was used in western blot analysis with commercial anti-Flag antibody, or the anti-ATP8A2 antibody. Both Flag (left panel) and ATP8A2 antibodies (right panel) detected a protein of the expected 130 kDa in lysates from *Atp8a2* cDNA transfected cells. (C) Total brain proteins were fractioned into membrane and cytosol fractions and subjected to SDS-PAGE and western blot analysis. ATP8A2 antibody detected the 130 kDa band only in the brain membrane fraction of wild type (*+/+*), *wl* (*Atp8a2^wl/wl^*) and *vmd* (*Atp8a2^vmd/vmd^*) mice. PSD95 was used as a membrane protein marker; TUBULIN was used as a marker for cytosolic proteins.

*Atp8a2* is widely expressed in the central nervous system including the cerebrum, cerebellum, spinal cord, and retina (Figure 7A). Ectopic expression of ATP8A2 in HEK-293T or COS7 cells showed that ATP8A2 has the expected molecular weight of a 130 kDa (Figure 7B), as detected by a polyclonal antibody against mouse ATP8A2 developed in our laboratory (see methods). Western blot analysis of protein isolated from either cytosolic or membrane fractions showed that ATP8A2 is localized to the mouse brain membrane fraction (Figure 7C).

### ATP8A2 is a phosphatidylserine translocase

**Figure 8:**
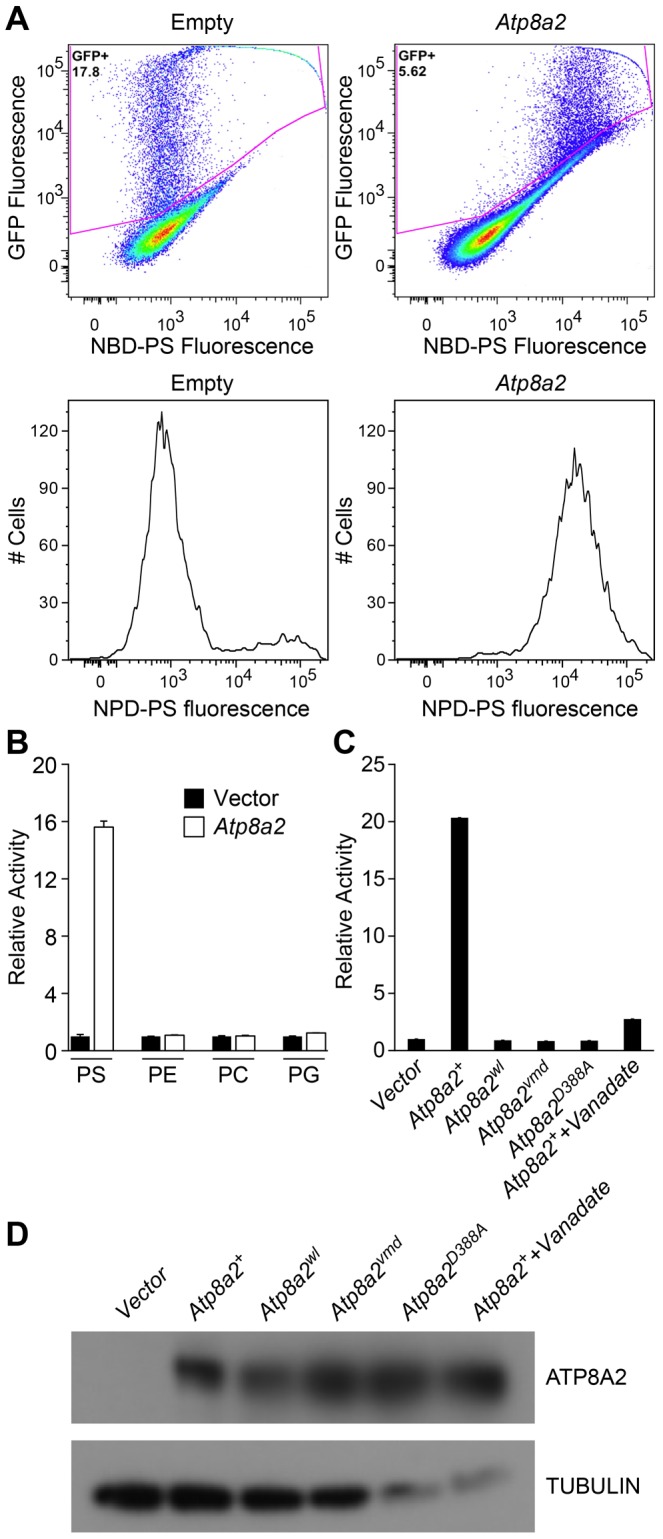
ATP8A2 contains phosphatidylserine translocase activity. (A) Internalization of NBD phospholipids by UPS-1 cells transfected with a control vector (Empty) or plasmid expressing *Atp8a2*. Expression of ATP8A2 leads to a population of UPS-1 cells with increased NBD-PS uptake (Top right panel). Representative numbers of NBD-PS-labeled UPS-1 cells are shown (Bottom panel). The X-axis represents NBD-PS fluorescence intensity of GFP-positive cells; cells transfected with pcDNA62-*Atp8a2* vector shows increased fluorescence intensity (bottom right panel), compared to cells transfected with empty vector (bottom left panel). (B) ATP8A2 specifically translocates NBD-PS across the plasma membrane of UPS-1 cells. Lipid translocation activity is shown as a percentage of NBD-lipid fluorescence intensity relative to control empty vector (defined as 1). Results are representative data from three independent experiments. No translocation activity was observed for NBD-phospholipids PE- phosphatidyletholamine, PC- phosphatidylcholine, or PG- phosphatidylglycerol. Four independent samples were assessed for each group. (C) Mutant proteins encoded by *wl* and *vmd* mutant mice are non-functional for PD translocation. Lipid translocation activity of *ATP8A2^wl^* and *ATP8A2^vmd^* encoded by *wl* and *vmd* mutant animals are similar to the vector control. A single D→A point mutation (*ATtp8a2^D388A^*) in the conserved DKLTG motif completely abolishes the lipid translocation activity of ATP8A2. A chemical ATPase inhibitor sodium vanadate significantly reduces ATP8A2 activity. (D) ATP8A2 protein levels were similar in all transfected cells (other than those transfected with empty vector) as assessed by western blotting analysis using the ATP8A2 antibody.

Based on sequence comparison, we hypothesized that ATP8A2 is likely a phospholipid translocase. To test this hypothesis, full-length *Atp8a2* cDNA was expressed in UPS-1 cells. These cells are defective in non-endocytic uptake of 7-nitrobenz-2-oxa-1,3-diazol-4-yl phosphatidylserine (NBD-PS) analogs and are thus optimal for testing phospholipid translocase (flippase) activity with NBD substrates [34], [35]. Compared to control cells transfected with empty vector, *Atp8a2* transfected UPS-1 cells displayed significant phosphatidylserine translocase activity (1500% of control, Figure 8A, B). This phospolipid flippase activity is specific to phosphatidylserine (PS), as translocation of only NBD-phosphatidylserine and not of NBD-phosphatidyletholamine (PE), NBD-phosphatidylcholine (PC) or NBD-phosphatidylglycerol (PG) was observed (Figure 8B). To ascertain that PS translocation was due to ATP8A2 activity, we generated a mutant version of ATP8A2 in which Asp388 in the highly conserved core sequence DKTGTLT was replaced with an Ala. This Asp residue is phosphorylated and dephosphorylated during the catalytic cycle and is critical for the activity of all P-type ATPases. The mutant protein encoded by *Atp8a2^D388A^* failed to translocate NBD-PS into the inner leaflet of the plasma membrane (Figure 8C). Similarly, the chemical inhibitor sodium vanadate greatly reduced the flippase activity of ATP8A2 (Figure 8C).

To examine the activity of the mutant protein encoded by the *wl* mutant alleles, we introduced the 21 bp deletion identified in the *wl* mutant, and the 108 bp deletion of the *vmd* mutant into the *Atp8a2* cDNA by site-direct mutagenesis. Although these mutant proteins were expressed (Figure 8D), neither protein displayed any flippase activity (Figure 8C). These data clearly demonstrate that the mutant proteins encoded by *wl* and *vmd* have no flippase activity.

## Discussion

Axon degeneration and axonopathies are often observed in human neurodegenerative diseases, but their molecular causes are typically not known. To provide new insight into axon degeneration we studied the wabbler-lethal (*wl*) mouse. We show that *wl* mutant mice develop distal axon degeneration and neuronal chromatolysis in varying parts of the CNS and the PNS without cell death. Together with our finding that the *Wld^s^* mutation, which protects against axon injury, significantly delayed axonal degeneration [25]–[30], [36]–[39], this provides strong evidence that the *wl* mutation induces an axonopathy [11]–[15].

Although the *Wld^s^* mutation delays axonal degeneration in *wl* mice, it does not alter their gross phenotype or extend lifespan. Similar to the effect of *Wld^s^* in *wl* mice, *Wld^s^* inhibits axonal spheroid pathology in gracile axonal dystrophy (*gad*) mice with the *Uchl1^gad^* allele, but did not alleviate *gad* symptoms [38]. Compared to *gad* mice, both the gracile nucleus and cervical gracile fascicle contained fewer spheroids in *Uchl1^gad^ Wld^s^* mice. However, similar to the previous observation that *Wld^s^* has a weaker effect on synapses than on axons, motor axon terminals at neuromuscular junctions continued to degenerate in *Uchl1^gad^ Wld^s^* mice. This might contribute to the fact that *Wld^s^* did not alleviate *gad* symptoms. In contrast, in *pmn* mice, another motor neuron disease model, *Wld^s^* was able to delay axonal degeneration, extend life span, and improve motor performance [25]. The fact that *Wld^s^* did not appear to alter the gross behavior of *wl* mice suggests that there may be detrimental phenotypes in *wl* mice that are distinct from axonal degeneration.

Two recent reports demonstrated a requirement of *Bax* during axonal degeneration [31], [32], and *Bax* deficiency delayed axonal degeneration in a mouse model of glaucoma [21]. In *wl* mice, genetic ablation of *Bax* significantly delayed axonal degeneration, providing the first evidence for the role of *Bax* in an inherited mouse model of primary axonal degeneration. Our result indicates that *Bax* has a role in intrinsic axon degeneration, similar to a previous study using cultured sensory neurons [31]. As in cultured sensory neurons [31], it is possible that caspase 6 participates in this process. This will be the subject of future investigations.

While the *wl* phenotype has been investigated for over 60 years, the underlying mutation was not previously identified. We show that mutations in *Atp8a2* underlie the phenotype for three independent *wl* alleles. *Atp8a2* encodes a protein homologous to P4-type ATPases, putative phospholipid translocases that translocate aminophospholipids from the extracellular leaflet to the cytoplasmic leaflet of the plasma membrane bilayer [40]. They are important for the maintenance of phospholipid asymmetry in eukaryotic cell membranes, and play essential roles in many physiological conditions. Our cell-based PS translocation assay clearly showed that ATP8A2 is a phosphatidylserine translocase and both the *wl* and *vmd* mutant proteins do not retain PS flippase activity. These findings are in agreement with recent independent studies showing that ATP8A2 has PS flippase activity when reconstituted in liposomes [41], [42].

The importance of the P4-ATPase membrane protein family is increasingly evident through findings in which dysfunction of P4-ATPases is associated with developmental defect in animals and several human disorders [43]–[47]. In *Caenorhabditis elegans* loss of the P4-ATPase TAT-1 leads to the exposure of PS on the surface of germ cells and loss of certain neuronal cells [48]. In humans, mutations in the *FIC1*/*ATP8B1* gene cause progressive familial cholestasis, a severe liver disease with defective bile secretion and hearing loss [43], [49]. Furthermore, *Atp8b3* is exclusively expressed in the testis and has a role in sperm capacitation in mice [50]. *Atp10a* (also named *Atp10c*) is linked to diet-induced obesity and type II diabetes in mice [51]. Mutations in the murine *Atp11c* gene leads to cholestasis and a striking B cell differentiation defect [45]–[47]. Although *Atp11c* is ubiquitously expressed in different tissues, loss of ATP11C activity specifically affects adult B cell development, indicating cell and lineage-specific requirement of this transporter.

Based on our findings, we propose a number of possible mechanisms by which loss of ATP8A2 activity can lead to axonopathy. Like ATP8B1, ATP8A2 is thought to be important for maintaining phospholipid asymmetry of cell membranes by translocating phosphatidylserine from the outer leaflet to the inner leaflet of the membrane. PS asymmetry in the cell membrane has been shown to have an essential role in the mechanical stability of the red cell membrane [52]. In patients and mice with an *ATP8B1/Atp8b1* deficiency, the canalicular membrane is not stable and extraction of lipid by the detergent action of bile salts leads to formation of granular bile and intrahepatic cholestasis. Similarly, loss of ATP8A2 activity may result in re-distribution of PS to the outer leaflet and loss of phospholipid asymmetry. Neuronal axon membranes may be unstable due to this abnormal PS distribution and thus become susceptible to degeneration. Alternately, loss of PS asymmetry might lead to a defect in intracellular sorting and transport of vesicular components similar to what was observed for the yeast homologue *Drs2*, which has a role in intracellular vesicular trafficking between the trans-Golgi network (TGN), the endosome and the plasma membrane [53].

Recent studies showed that disruption of phospholipid turnover and trafficking can lead to neurodegenerative diseases in both mice and people. For example, the spontaneous null mutation of mouse *Fig4* in the *pale tremor* (*plt*) strain leads to neuronal loss, spongiform degeneration of the brain and loss of neurons from the dorsal root ganglia [54], [55]. Mutant animals had a severe peripheral neuropathy and a shorter life span. *Fig4* encodes a phospholipid phosphatase with 5-phosphatase activity towards the 5-phosphate residue of PtdIns(3,5)*P*_2_ [56], [57]. Loss of the FIG4 phosphatase in humans leads to the autosomal recessive, demyelinating, Charcot-Marie-Tooth neuropathy (CMT), CMT type 4J (OMIM #611228) [54], [58]. The most common human mutation of *FIG4* reduces the binding affinity of FIG4 for the PtdIns(3,5)*P*2 biosynthetic complex [59], while in mouse *plt* fibroblasts a significant decrease of PtdIns(3,5)*P*_2_ was observed [54]. Intracellular phosphoinositides (PIs) are essential regulators of membrane trafficking, including functions to promote recruitment and/or activation of spatially localized protein machinery on membranes. The production and turnover of PIs are tightly controlled by kinases and phosphatases [60], [61]. In the nervous system, neurons, especially neurons with long axons, depend on efficient membrane trafficking for maintenance of proper functions and health [62]. In humans and mice, autosomal recessive, demyelinating, Charcot-Marie-Tooth type B1 neuropathy [63]–[65] is also caused by an abnormality of phospholipid metabolism resulting from mutation of the myotubularin-related 2 gene (*MTMR2*). MTMR2 is a phospholipid phosphatase with 3-phosphatase activity towards the 3-phosphate residue of PtdIns(3,5)*P*2 and PtdIns3*P* [66], [67]. Therefore, *Fig4*, *MTMR2* and *Atp8a2* mutations may affect neurons by altering membrane protein trafficking. These mutant phenotypes highlight the importance phospholipid metabolism for neuronal health and the possible role of abnormal membrane trafficking in neurological diseases.

Loss of lipid asymmetry has an important impact on cell morphology, membrane protein activities, phagocytosis, apoptosis, endocytosis, and vesicle biogenesis [68]. In addition proper membrane lipid composition is also important for exocytosis and synaptic vesicle release in neuronal cells. PS content has been shown to affect PC12 cell exocytosis [69]. Altered PS distribution in *wl* neurons could affect release of synaptic vesicles and transduction of action potentials along nerve fibers.

*Atp8a2* is specifically expressed in the nervous system and testis. In contrast, *Atp8a1*, a PS translocase and a close member of the P-type ATPase family, is expressed broadly in many tissues. *Atp8a1* deficient mice have no grossly visible neurological phenotypes and have grossly normal brains ([70] and www.informatics.jax.org/external/ko/deltagen/1902.html). Recently, behavioral analysis of *Atp8a1* deficient mice detected neurological abnormalities, including impaired hippocampus-dependent learning (Morris Water Maze test), hyperactivity, and poor maternal behavior [70]. Furthermore, deficiency of both *Atp8a2* and *Atp8a1* results in neonatal lethality (our unpublished data using the *wl* allele). Double mutant mice have labored breathing and die within a few hours after birth (unpublished observations). It is likely that ATP8A2 and ATP8A1 act redundantly in certain tissues to allow survival of single mutants by maintaining an adequate PS asymmetry. Consistent with this, no obvious neuronal death was detected in *Atp8a2^wl/wl^* mutants. Additionally, we could not detect any obvious disturbance of PS lipid asymmetry in cultured neurons and spermatogonia from these *Atp8a2^wl/wl^* mutants (not shown). Obvious loss of asymmetry has been well established to induce apoptosis [71]. Loss of both ATP8A2 and ATP8A1 leads to failure of tissue function and lethality. Conditional alleles of *Atp8a2* and *Atp8a1* will be helpful to pinpoint the temporal and tissue specific requirements of *Atp8a2* and *Atp8a1*.

Interestingly, due to a de novo chromosome translocation, a patient with mental retardation and hypotonia was haploinsufficient for *ATP8A2*. No other genes were reported to be structurally altered by this genetic event. However, no gene expression studies for *ATP8A2* or other genes flanking the translocation, whose expression may be affected by the translocation, were presented. Additionally, no mutations were found in thirty-eight other patients with similar phenotypes [72]. Thus, it is not yet clear if ATP8A2 plays a role in this disease. However, this patient together with the data presented in our current paper suggests that *ATP8A2* should be further considered as a candidate gene for human neurological diseases.

In conclusion, normal ATP8A2 activity is indispensable for normal neuronal functions. To our knowledge, ATP8A2 is the first mammalian PS flippase identified to have a role in axon degeneration. Our experiments identify a new process that contributes to axonopathy and neurological disease.

## Materials and Methods

### Mouse strains and genotyping

The Association for Assessment and Accreditation of Laboratory Animal Care guidelines were followed for all animal procedures, and all procedures were approved by the Institutional Animal Care and Use Committee of The Jackson Laboratory. The original *wabbler-lethal* (*wl*) mutant arose in the *pirouette* strain of *Mus musculus* at The Jackson Laboratory [11]. The *Vestibulo-Motor Degeneration* (*vmd*) mutation arose in a C3H/H2SnJ colony at the Jackson Laboratory in 1987. Both *wl* and *vmd* mice were obtained from The Jackson Laboratory and backcrossed to C57BL/6J (B6) for at least ten generations. Before PCR based genotyping protocols were established, heterozygous mice of each strain (*wl* and *vmd*) were crossed and matings producing homozygous *wl* or *vmd* mutants were kept for strain production. As has become standard practice for these mutant mice, dry food was supplemented with a soft maintenance diet (DietGel 76A, ClearH_2_O). After identifying the mutations in *Atp8a2* in the *wl* and *vmd* strains, PCR based protocols were established for genotyping. For *wl*, the primer pair wl-L 5′ - TGAACTGTCCCTTAACTGATGGTA - 3′ and wl-R, 5′ - TGGCTATGGTTTCTGGAACG - 3′ (Figure 6) were used. This primer pair spans the 21 base-pair deletion in exon 22 in *wl* mutant mice and produces a 108 bp amplicon in wild type controls, and an 87 bp amplicon in *wl/wl* mice. For the *vmd* genomic deletion, three primers flanking the region were used (Figure S10) to distinguish between the wild type and mutant alleles: vmdF, 5′ - CTAACTGTGGCTCACTTACCTCCT - 3′; vmdR1, 5′ - TCCTCCAGAACATTGAAGTGACTA - 3′; vmdR2, 5′ - TGCATCTTGATTTTTGCTTTGTAT - 3′. A 403 bp amplicon is produced in the presence of the wildtype allele using primer vmdF and vmdR1 and a 207 bp amplicon is produced in the presence of the mutant *vmd* allele using primers vmdF and vmdR2. *wl^3J^* arose in the CBA/J production colony at The Jackson Laboratory [73]. This strain is cryopreserved, and therefore genomic DNA of *wl^3J^* mutant and control animals was obtained from the DNA Resource at The Jackson Laboratory to determine the mutation.

To assess the effect of the *Wld^s^* allele on axonopathy in *wl* mice, the original *Wld^s^* allele (*Wld^s^*) [26]–[29], [74] was obtained from Harlan Sprague Dawley on a C57BL/10Hsd background. This allele is maintained by continuously backcrossing to C57BL/6J and was crossed to *wl* mice to generate the *wl Wld^s^* strain. All experiments assessing the effect of *Wld^s^* on axon degeneration were performed using mice hemizygous for *Wld^s^*. A *Bax* null allele (B6.129X2-*Bax^tm1Sjk^*/J, herein referred to as *Bax* ^−^) was obtained from The Jackson Laboratory and crossed to *wl* mice to generate *wl Bax* ^−/−^ mice.

### Histology

Anesthetized mice were fixed by transcardial perfusion with physiological saline (PBS), followed by freshly made 2% glutaraldehyde and 2% paraformaldehyde in 0.1 M cacodylate buffer (pH 7.4). Sciatic and femoral nerves were dissected under a dissecting microscope and post-fixed overnight in the same fixative. After post-fixing, nerves were rinsed twice with PBS and processed for plastic embedding, histological staining and transmission electron microscopy (TEM) by standard procedures [75]. Briefly, 0.5 µm semi-thin plastic sections were stained with toluidine blue and examined by light microscopy. The total number of myelinated axons in each nerve was counted using toluidine blue-stained plastic sections and a Leica DMRE microscope. For TEM, nerves were treated with uranyl acetate and standard embedding was done as previously described [75]. TEM images were collected on a Jeol 1230 microscope. Axon diameters of P30 and P60 mice were determined from six non-overlapping 5000× fields from each of three mutant and three littermate control samples. Axon diameters were measured using the associated software AMT Image Capture Engine. For axon counts, left and right nerves were taken, and counts obtained for both nerves were averaged. Thus each count sample represents the average count of the left and right nerve of one mouse.

For histological analysis of brain and spinal cord sections, anesthetized mice were fixed by transcardial perfusion with 2% glutaraldehyde and 2% paraformaldehyde in 0.1 M cacodylate buffer (pH 7.4). Tissues were dissected out, embedded in paraffin, serial sections obtained and stained using hematoxylin and eosin (H&E). To assess morphological structures of the retina, enucleated eyes were fixed overnight in 1.2% glutaraldehyde and 0.8% paraformaldehyde in 0.08 M phosphate buffer, embedded in Technovit resin, cut in 1.5 µm sections and stained with hematoxylin and eosin (H&E).

For analysis of the optic nerve sections with PPD staining, intracranial portions of optic nerves were processed and analyzed as previously described [19]–[23]. Briefly, optic nerves were fixed *in situ* in 1.2% glutaraldehyde and 0.8% paraformaldehyde in phosphate buffer for 48 hours, dissected free, processed, and embedded in plastic. One-micron-thick cross sections of optic nerve from behind the orbit were cut and stained with paraphenylenediamine (PPD). PPD darkly stains the myelin sheaths and axoplasm of sick or dying axons but not healthy axons.

### Immunohistochemistry

For immunohistochemistry enucleated eyes or carefully dissected free spinal columns were fixed overnight in 4% paraformaldehyde in PBS, and then cryoprotected in sucrose. Tissues were embedded in optimal cutting temperature solution (OCT) and frozen on dry ice for sectioning. Neurofilament was stained with pNF antibody (Covance, 1∶500) and visualized with AlexaFluor488-conjugated anti-mouse IgG (Invitrogen). Nuclei were counterstained with DAPI (Sigma). Active caspase-3 (1∶200) was purchased from R&D Systems. Sections were analyzed and imaged on a Leica TCS SP5 II confocal microscope. Anti-Tubulin antibody was obtained from Sigma and used at a dilution of 1∶2000. Mouse anti-PSD95 was obtained from Neuromab (http://neuromab.ucdavis.edu/) and used at a dilution of 1∶1000.

### Mapping the *wl* mutation

The *wl* mutation had originally been mapped to chromosome 14, close to the hairless (*hr*) locus [33]. To further narrow the interval, *wl*/+ male mice originally obtained from The Jackson Laboratory on a mixed background were crossed to C57BL/6J wild type females to generate F1 progeny. F1 progeny were intercrossed to generate F2 animals. Offspring from these intercrosses were examined at 3 weeks of age to identify those with abnormal gait and thus homozygous for the *wl* allele. Tail tissue was obtained from a total of 688 affected animals for DNA preparation and analysis. To narrow the region, MIT markers able to distinguish between C57BL/6J and *wl* on chromosome 14 were used [i.e. D14Mit154 (58.6Mb), D14Mit113 (60.4 Mb), DLM14-10 (60.6 Mb), DLM14-21 (61.4 Mb), D14Mit3 (61.5 Mb), and D14Mit37 (63.0 Mb)]. Primer sequences are listed in Table S1.

### Identifying the mutation

Using the mapping strategy describe above, the region was narrowed to an interval of 773 Kb containing 10 genes. Primer pairs were designed to span the coding exons and splice sites for all 10 genes in the critical region. Those designed for *Atp8a2* are given in Table S2. These primer pairs were utilized to obtain PCR products using Abgene 2X ReddyMix PCR Master Mix (1.5 mM MgCl_2_) with genomic DNA from *wl/wl* mutants as well as littermate controls as template. PCR products were purified using the QIAquick PCR Purification Kit (Qiagen) and subjected to sequencing using the BigDye Terminator Cycle Sequencing Kit (Applied Biosystems). Sequences were analyzed using the Sequencher 4.2 software, comparing publically available sequences for *Atp8a2* with the sequences obtained for mutant and control mice. The same primer pairs and strategy were used to determine the mutation in *wl^vmd^* and *wl^3J^* mice.

### Molecular cloning and site-directed mutagenesis of the *Atp8a2* open reading frame (ORF)

Full-length cDNA of *Atp8a2* (NM_015803.2) was synthesized using the de-novo DNA synthesis technique (GenScript Corporation, NJ, U.S.A.) and cloned into pUC57 with two flanking restriction enzyme sites *EcoRV* and *SalI*. The ORF itself is also flanked with *AscI* and *MluI* sites. The *Atp8a2* ORF was sub-cloned into the pCMV6-AN-Flag (Origene) expression vector using the *AscI* and *MluI* restriction endonuclease sites to produce pCMV6-AN-Flag-ATP8A2. All sequences were confirmed using sequence analysis.

For the lipid translocation assays, the *Atp8a2* ORF was amplified by PCR and sub-cloned into the pEntry1A dual selection vector (Invitrogen) at *NotI* and *XhoI* sites to generate pEntry1A-*Atp8a2*. PCR primers used were: Atp8a2-NotI: 5′-CACCAGTCCCGGGCCACGTCTGTTGGAGACC-3′; Atp8a2-XhoI: 5′-TTATTTCTTCCTTTCTCGAGTCTTTGGTGGTATCATAAGCGC-3′. The destination vector pcDNA6.2/EmGFP-Bsd/V5-DEST (Invitrogen Catalog no. V366-20) was used to generate the expression vector pcDNA6.2-*Atp8a2* by performing an LR recombination reaction following the manufacture's instruction. pcDNA6.2/EmGFP-Bsd/V5-DEST contain the human cytomegalovirus immediate-early (CMV) promoter/enhancer for high-level expression of the gene of interest and the murine phosphoglycerate kinase-1 (PKG) promoter to drive expression of the Emerald GFP-Blasticidin (EmGFP-Bsd) fusion protein in mammalian cells. Transfected cells express both ATP8A2 and EmGFP-Bsd and can be detected by flow cytometry.

*pcDNA6.2-Atp8a2^wl^* was generated by introducing a 21 bp deletion into pcDNA6.2-*Atp8a2* vector using the QuickChange II XL Site-Directed Mutagenesis Kit (Stratagene) according to manufacturer's instructions. Primers: wl-21bpdel-F, 5′-CTGTTACTTGGAG CTACAGCCGGCGTTCCAGAAACCATAGCCACTC-3′; wl-21bpdel-R, 5′-GAGTGGCTATGGTTTCTGGAACGGCTGTAGCTCCAAGTAACAG-3′. Similarly, pcDNA6.2-*Atp8a2^D388A^* was generated by the introduction of Asp→Ala (GAC→GCC) mutation into the pcDNA6.2-*Atp8a2* vector. Primers: Atp8a2-changeD-F, 5′-GGGCAGGTAAAATACCTGTTTTCAGCCAAGACTGGAACTCTTACATGT -3′; Atp8a2-changeD-R, 5′-ACATGTAAGAGTTCCAGTCTTGGCTGAAAACAGGTATTTTACCTGCCC -3′. pcDNA6.2-*Atp8a2^vmd^* was generated by swapping the cDNA fragment (basepair 1489–3447th ) with the corresponding fragment amplified from *vmd/vmd* brain cDNA (HindIII and XhoI sites of pEntry1A-*Atp8a2*). Primers: vmdP1, 5′-AGCTAAGAAGCTTGGCTTTGTGTTTACCGGGAGG-3′; vmdP2, 5′-TTATTTCTTTGAATTCTCTTTGGTGGTATCATAAGCGC-3′. All mutant versions of *Atp8a2* were confirmed by sequencing.

### RT–PCR

Tissue from 1–2 months old animals were dissected free and placed into RNAlater (Ambion) at room temperature. Total RNA was prepared from these tissues using TRIzol Reagent (Life Technologies) according to the manufacturer's instructions. RNA samples were treated with RNase-free DNaseI (Ambion) and RNA concentration determined using a NanoDrop (ND-1000) spectrophotometer. 10 µg RNA was reverse transcribed using random primers and the MessageSensor RT Kit (Ambion). The primers used for PCR were: *Atp8a2*, Atp8a2F1 = 5′-GGAGCAGATCCTGGAGATTGACT-3′; Atp8a2R1 = 5′-CGGAAGCACTCTC-3′; *beta-Actin*, ActinbF1 = 5′-AGCCATGTACGTAGC CATCC-3′; ActinbR1 = 5′-CGGCCAGCCAGGTCCAGAC-3′.

### Antibody production

The ATP8A2 antibody was produced by Proteintech Group, Inc (Chicago, IL) using an ATP8A2-GST fusion protein designed in our laboratory. Briefly, a cDNA fragment encoding amino acid 536–840 of the ATP8A2 P-domain was amplified using the following primers: Atp8a2-PDF1, 5′-TTTTGGATCCATGTCTGTCATTGTCCGACTG-3′, Atp8a2-PDR1, 5′- TTTTCTCGAGTCAGTACAGGATACACTTGGTC-3′. The resulting PCR product was cloned in-frame with glutathione S-transferase (GST) in the pGEX-4T-1 vector (GE healthcare) using BamHI and XhoI restriction enzymes. The resulting plasmid was validated by sequencing and transformed into *E. coli*. BL21 was used for ATP8A2 fragment-GST fusion protein production. The fusion protein was purified with glutathione-Sepharose (GE Healthcare) using standard procedures. The purified fusion protein was used for immunization of rabbits. The ATP8A2 antibody was purified from serum by affinity purification using the GST-ATP8A2 fragment fusion protein.

### Expression of *Atp8a2* in HEK-293T cells

HEK-293T cells (American Type Culture Collection (ATCC), Manassas, VA) were cultured in DMEM medium with high glucose (HyClone) supplemented with 10% fetal bovine serum and 1% (vol/vol) penicillin/streptomycin at 37°C in a 5% CO_2_ atmosphere. Cells were seeded in six-well plates (Corning, NY) and transfected at 50% confluency with 1 µg of pCMV6-AN-Flag-*Atp8a2* or empty vector using Lipofectimine 2000 (Invitrogen) according to the manufacturer's instructions. Cells were harvested after 48 hours.

### UPS-1 cell culture

The CHO UPS-1 cell line is a mutant Chinese hamster ovary (CHO) cell line defective in the nonendocytic uptake of NBD-PS [34], and was kindly provided by Dr. Robert Pagano from Mayo Clinic (Rochester, MN). UPS-1 cells were grown in Ham's F12 medium supplemented with 10% fetal bovine serum and 1% glutamine (vol/vol), and 1% (vol/vol) penicillin/streptomycin at 37°C in a 5% CO_2_ atmosphere. Cells were seeded in 6-well plate and allowed to grow to 50% confluency. Transfection with empty vector or plasmid pcDNA62-Atp8a2 expressing ATP8A2 was carried out using Lipofectamine 2000 (Invitrogen) according to the manufacturer's instructions.

### Lipid translocation assay

The following labeled phospholipid analogs were purchased from Avanti Polar Lipids: 16:0–06:0 NBD-PS [1-palmitoyl-2-[6-[(7-nitro-2-1,3-benzoxadiazol-4-yl)amino] hexanoyl]-sn-glycero-3-phospho-L-serine (ammonium salt)]; 16:0–06:0 NBD-PE [1-palmitoyl-2-[6-[(7-nitro-2-1,3-benzoxadiazol-4-yl)amino] hexanoyl]-sn-glycero-3-phospho-L-etholamine (ammonium salt)]; 16:0–06:0 NBD-PC [1-palmitoyl-2-[6-[(7-nitro-2-1,3-benzoxadiazol-4-yl)amino] hexanoyl]-sn-glycero-3-phospho-choline (ammonium salt)]; and 16:0–06:0 NBD-PG [1-palmitoyl-2-[6-[(7-nitro-2-1,3-benzoxadiazol-4-yl)amino] hexanoyl]-sn-glycero (ammonium salt)]. NBD-lipid powder stocks were dissolved in 95% ethanol and diluted to 20 µM with Hank's balanced salt solution with 15 mM MgCl_2_ and without phenol red (HBSS-15 mM MgCl_2_; Gibco). The translocation of NBD-lipid was determined as described with some modification [35]. In short, transfected UPS-1 cells grown to confluency in 6-well plates (Corning, NY) were washed, equilibrated in pre-warmed HBSS-15 mM MgCl_2_ for 15 minutes at 20°C, and incubated with 20 µM NBD-lipid for 20 minutes at 20°C. Subsequently, the cells were washed with HBSS-15 mM MgCl_2_ on ice. To quantify NBD-lipid translocated into the inner leaflet of the plasma membrane, lipid from the outer leaflet was removed by back-extraction. This was done by adding ice-cold HBSS supplemented with 2% bovine serum albumin (Sigma) to the cells for 10 min on ice, and repeating the process three times. Finally, the cells were washed with cold HBSS, treated with 0.25% Trypsin and suspended in HBSS. One microliter of 5 mg/ml DAPI in PBS was added to 3×10^6^ cells in 0.5 ml HBSS just before FACS analysis. Flow cytometry of NBD-lipid labeled UPS-1 cells were performed on a Becton Dickinson LSR II cytometer equipped with an argon laser using FACSDiVa software. Ten thousand GFP positive cells were analyzed during the acquisition. Dead cells were excluded from the analysis by blue fluorescence (DAPI positive). The data were analyzed with FlowJo software (Tree Star Inc., Ashland, OR). The NBD-lipid fluorescence intensity of living UPS1 cells was plotted on a histogram to calculate the median fluorescence intensity. Lipid translocation activity was calculated as a ratio to vector control samples.

### Western blotting

Brain and spinal cord were collected from 1- to 2-month old mice and put directly into lysis buffer (10 mM Tris-HCL, pH 7.4, 100 mM NaCl, 1.5MgCl_2_, plus Complete Protease Inhibitor Cocktail (Roche)). Tissues were homogenized using a motor-driven Polytron homogenizer and the resulting lysates were centrifuged at 500 g to remove intact cells. To separate the cytosol and membrane fractions, the supernatant was subjected to centrifugation at 100,000 g for 45 min at 4°C using a Beckman SW40 rotor. The supernatant was collected as the cytosolic fraction and the pellet was resuspended in lysis buffer plus 0.1% Triton X-100 on ice for 20 min as membrane fraction. Total protein concentration was determined using the BCA Protein Assay Reagent (Pierce). Equal amounts of cytosol and membrane proteins were separated by electrophoresis using a 7.5% Mini-PROTEAN TGX Precast Gel (BioRad). Proteins were transferred to PVDF membranes (GE healthcare) and detected by immunoblotting using standard techniques.

### Conduction Velocity (CV) recording

Conduction velocity of sciatic axons was determined by measuring the latency of compound motor action potentials recorded in the muscle of the left rear paw [76]. Mice were anesthetized with 1%–1.5% isoflurane and placed on a thermostatically regulated heating pad to maintain normal body temperature. Action potentials were produced by subcutaneous stimulation at two separate sites: proximal stimulation at the sciatic notch and by a second pair of needle electrodes placed distally at the ankle. For recording, a needle electrode was inserted in the center of the paw (active) and a second electrode was placed in the skin between the first and second digits. Conduction velocity was calculated as [(proximal latency – distal latency)/conduction distance]. Four animals of each genotype were tested.

### Wire hang test

For the wire hang test each animal was placed on a wire cage top held approximately a foot above an empty cage. The wire cage top was then gradually inverted and the time each mouse was able to hang onto the cage top was recorded. If any mouse still gripped the wire top after 60 seconds, the animal was removed and the time was recorded as 60 seconds.

### Statistical analysis

All data are presented as means ± SEM. Data were analyzed in JMP7.0 (SAS Campus Drive, Building T, Cary, NC). The differences between two groups was assessed by Student's *t*-test; *p*<0.05 was considered statistically significant.

## Supporting Information

Figure S1 Chromatolysis in the medial cerebellar nucleus and the lateral vestibular nucleus. (A, B) Hematoxylin and eosin staining of the medial cerebellar nucleus in wild type (*+/+*) and *wl/wl* mice; white arrows indicate neurons with characteristics of central chromatolysis. (C, D) Hematoxylin and eosin staining of the lateral vestibular nucleus in wild type (*+/+*) and *wl/wl* mice; white arrows indicate neurons with characteristics of central chromatolysis. Scale bar is 50 µm.
(TIF)

Figure S2 Caspase 3 activity is not increased in *wl/wl* mice. Cleaved caspase 3 staining of the lateral cerebellar nucleus (DCN), intermediate reticular nucleus (IRT) and spinal cord. No caspase 3 staining was detected in *wl/wl* animals in the DCN (A, B), the IRT (C, D) or the spinal cord (E, F). Positive control (G): cleaved caspase 3 staining of the cerebellar granule cell layer in a wild type animal at ten days of age when some granule cells die through an apoptotic mechanism during the developmental process (red – indicated by arrows). Blue staining is nuclei stained with DAPI. Scale bar is 50 µm.
(TIF)

Figure S3 Dystrophic axons in the corticospinal and spinalcerebellar tracts. (A, B) Hematoxylin and eosin staining of the cortocospinal tract in wild type (*+/+*) and *wl/wl* mice at two months of age; arrows indicate dystrophic axons. (C, D) Hematoxylin and eosin staining of the spinalcerebellar tract in wild type (*+/+*) and *wl/wl* mice; arrows indicate dystrophic axons. Scale bar is 50 µm.
(TIF)

Figure S4 Dystrophic axons in the spinal cord. (A, B) Luxol fast blue staining, another stain that allows for identification of dystrophic axons, of spinal cord sections in wild type (*+/+*) and *wl/wl* mice. Arrows indicate dystrophic axons. Scale bar is 50 µm.
(TIF)

Figure S5 Progressive loss of motor axons in the femoral nerve in *wl/wl* mice. (A–F) Semi-thin (1 µm) sections of the motor branch of the femoral nerve were stained with toluidine blue and examined for axonal degeneration. There was no obvious difference in the number of axons between mutants and controls at eight days after birth (P8), but there was axons loss in *wl/wl* nerves at thirty (P30) and sixty (P60) days after birth. (G) Myelinated axons from sections obtained at different ages were counted in control and *wl/wl* mice. Axons in two sections per mouse for 4 mice of each age and genotype were counted. At P8, no significant change in axon number was found in the motor branch of the nerve in control (495±7) and *wl*/*wl* (484±3) mice. However, at P30 a significant decrease in the number of myelinated motor axons was found, with 534±3 axons counted in control mice versus to 449±4 axons counted in *wl/wl* mice (p<0.01). At P60, significant additional loss of axons was evident in the motor branch with 530±34 axons in wild type control mice versus 397±4 in *wl/wl* mice (p<0.01). The values are means of axon number in each nerve ± SEM. Scale bar is 50 µm.
(TIF)

Figure S6 Loss of large diameter axons in the sciatic nerve. (A) Representative semi-thin (1 µm) sections of sciatic nerves obtained from 3 control and 3 *wl/wl* mice at P30 and P60 were stained with toluidine blue. At P30, sciatic nerves from control (*+/+*) and mutant (*wl/wl*) mice were indistinguishable, but at P60 less large diameter axons were present in mutant than in control mice. (B) At P30, the distribution of axonal diameters is similar in control (*+/+*; with a mean value of 3.45±0.07 µm) and mutant mice (*wl/wl*; with a mean of 3.62±0.09 µm). A total of 232 axons from three control mice and 333 axons from 4 mutant mice were measured. (C) In contrast, at P60 the mean axonal diameter is decreased from 4.70±0.11 µm in controls to 3.31±0.06 µm in mutants. Consistent with the loss of large-diameter axons, myelin thickness is reduced in mutant mice from a mean of 0.99±0.02 to 0.57±0.01 (p≪0.01, 333 axons from three P60 *+/+* mice and 399 axons from four P60 *wl/wl* mice were analyzed by TEM). Scale bar is 50 µm.
(TIF)

Figure S7 Axon transport defects - Phosphorylated neurofilament (pNF) accumulates in soma of neurons in the cerebellar nucleus, intermediate reticular nucleus and raphe magnus nucleus. (A, B) pNF localized to only the axons in the medial cerebellar nucleus (medial DCN) in wild type mice. It was not present in the soma. In *wl/wl* mice and indicative of an axon transport defect, however, pNF had accumulated in the somas of neurons of the medial cerebellar nucleus. Similarly, pNF also accumulated in the somas of neurons in the intermediate reticular nucleus (IRT; C, D) and the raphe magnus nucleus (RMG; E, F) of *wl/wl* mice but not control mice. Tissues were collected from animals at two months of age. Scale bar is 50 µm.
(TIF)

Figure S8 Example of axonal degeneration in the optic nerve detected by PPD staining. (A, B) Representative images of wild type B6 (A) and *wl/wl* (B) optic nerve semi-thin sections stained with PPD. Optic nerves are from mice at P60, an age where a low level of axonal degeneration is present in *wl/wl* mice. Arrows indicate damaged axons that stain darkly with PPD. Scale bar is 50 µm.
(TIF)

Figure S9 The *Wld^s^* gene does not improve performance of *wl/wl* mice in the wire hang test. In the wire hang test, *wl/wl* and *wl/wl Wld^s^* mice were able to grip the cage top for an average of 12.2±3.4 and 13.2±2.9 seconds respectively (P>0.05), while wild type controls gripped for an average of 55.2±3 seconds.
(TIFF)

Figure S10 Characterization of the *vmd* mutation. (A) Diagram showing the structure of the *Atp8a2* genomic locus and location of the *vmd* deletion (indicated by a purple triangle). Primers used for PCR genotyping are indicated by arrows. (B) Sequencing trace showing the deletion in *vmd* genomic DNA. Deleted sequence is underlined. Nucleotide bases immediately next to the deletion are boxed. *vmd 5*′ break point: 60,391,594 bp; 3′ break point: 60,382,428 bp. (C) Sequence analysis of *vmd* cDNA revealed the loss of exon 32 in *vmd* cDNA. The base pairs deleted by the *vmd* mutation are underlined, while nucleotide bases immediately flanking the deletion are boxed. (D) PCR based genotyping method used to detect the 9,167 bp deletion in *vmd* genomic DNA. A band of 403 bp is amplified in control mice using primer F and R1 (+/+). In contrast, a 207 bp product is amplified using primers F and R2 in mutant mice (*vmd/vmd*). In mice heterozygous for *vmd* (*vmd/+*), both products are amplified. (E) Detection of *vmd* deletion in cDNA by PCR using two primers flanking the deleted region. A product of 272 bp is amplified in *vmd* cDNA. In contrast, a 380 bp band is observed in control cDNA.
(TIF)

Figure S11 Characterization of the *Atp8a2^wl3J^* mutation. (A) Diagram showing the structure of *Atp8a2* genomic locus with the *3J* mutation indicated using a red triangle (deletion) and rectangle (duplication) respectively. Primers used for PCR genotyping are represented by two arrows. (B) Sequencing trace showing the genomic break points flanking the *3J* mutation. The nucleotide bases immediately 5′ to the 641 bp deletion are boxed. (C) The genomic duplication in exon 32 in *3J*. The sequence TCTTTGGTGT, which is boxed, is duplicated in *3J* DNA. (D) Result of a PCR based genotyping method to detect the 641 bp deletion in *3J* genomic DNA using the primers indicated in (A). A band of 1003 bp is amplified in control mice using primer 3J–F and 3J–R. In contrast, a 362 bp product is amplified using the same primer pair with *3J* genomic DNA as a template.
(TIF)

Table S1 DNA primers for mapping *wl*.
(XLS)

Table S2 DNA primers for sequencing *Atp8a2* cDNA.
(XLS)
