# Supplementary figures and images for: Mutations in a P-Type ATPase Gene Cause Axonal Degeneration
Source: PLoS Genet. 2012 Aug 9;8(8):e1002853. doi: 10.1371/journal.pgen.1002853 (PMC3415440; doi:10.1371/journal.pgen.1002853)

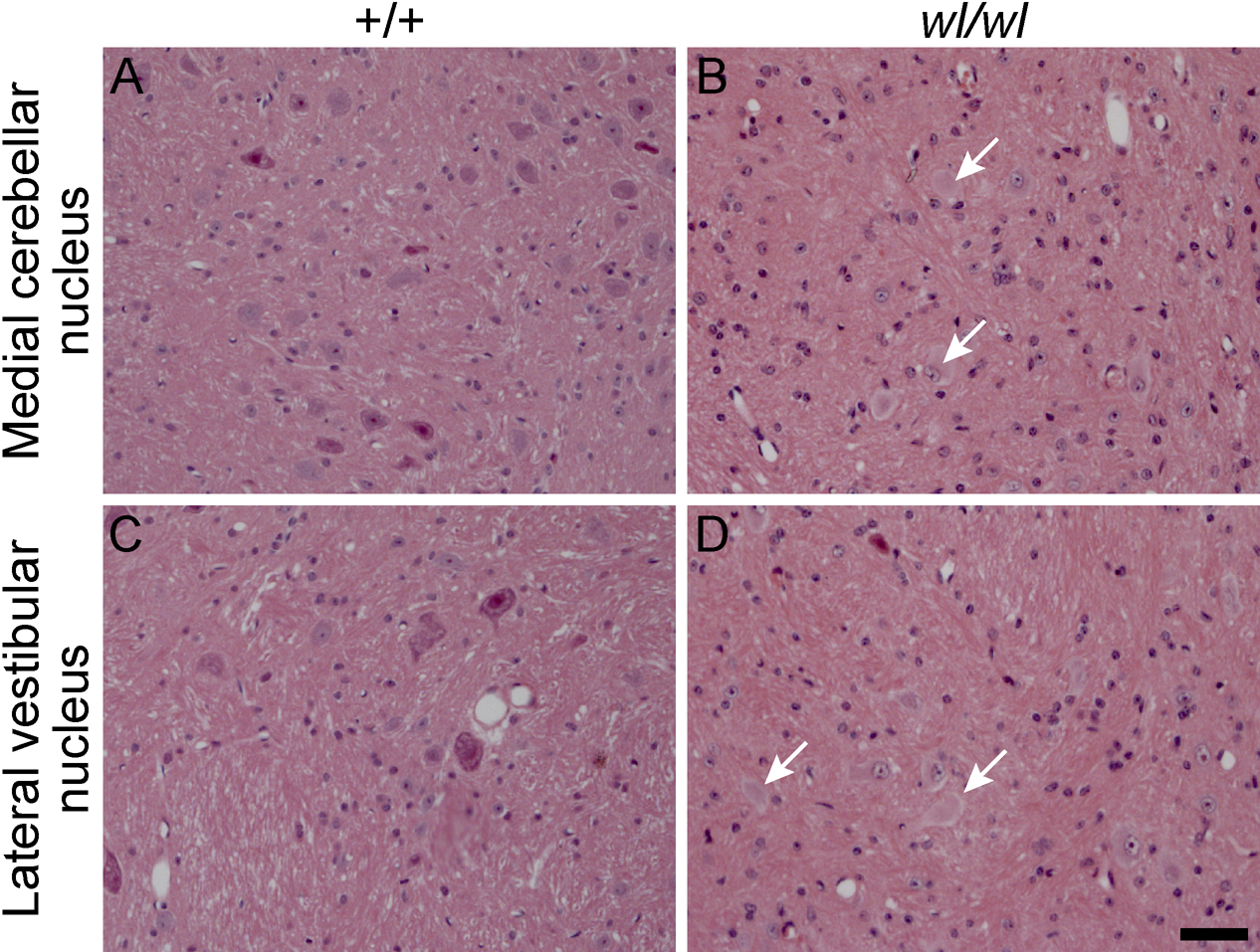

Supplement: Figure S1 — Chromatolysis in the medial cerebellar nucleus and the lateral vestibular nucleus. (A, B) Hematoxylin and eosin staining of the medial cerebellar nucleus in wild type (+/+) and wl/wl mice; white arrows indicate neurons with characteristics of central chromatolysis. (C, D) Hematoxylin and eosin staining of the lateral vestibular nucleus in wild type (+/+) and wl/wl mice; white arrows indicate neurons with characteristics of central chromatolysis. Scale bar is 50 µm. (TIF) [file pgen.1002853.s001.tif]

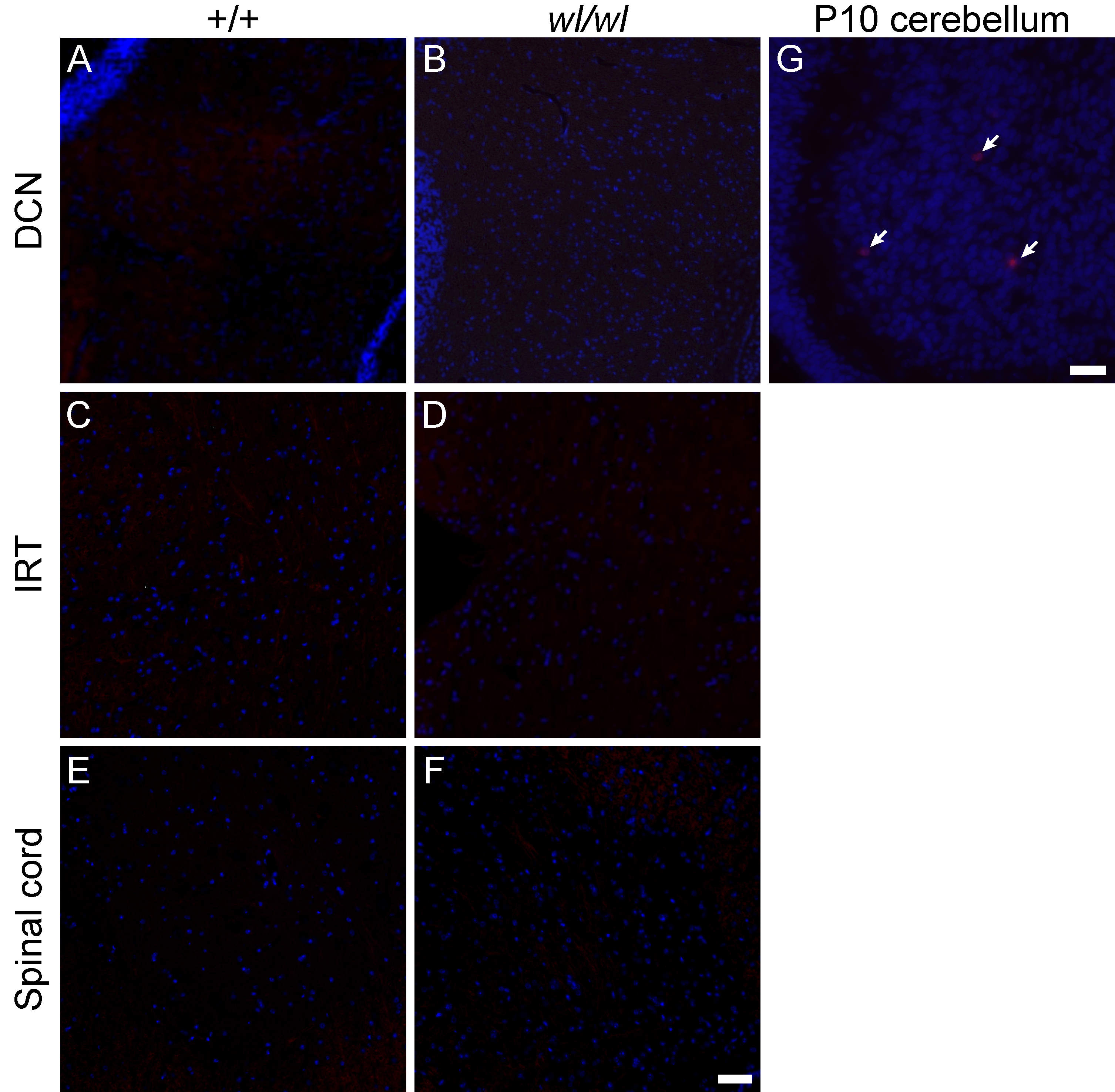

Supplement: Figure S2 — Caspase 3 activity is not increased in wl/wl mice. Cleaved caspase 3 staining of the lateral cerebellar nucleus (DCN), intermediate reticular nucleus (IRT) and spinal cord. No caspase 3 staining was detected in wl/wl animals in the DCN (A, B), the IRT (C, D) or the spinal cord (E, F). Positive control (G): cleaved caspase 3 staining of the cerebellar granule cell layer in a wild type animal at ten days of age when some granule cells die through an apoptotic mechanism during the developmental process (red – indicated by arrows). Blue staining is nuclei stained with DAPI. Scale bar is 50 µm. (TIF) [file pgen.1002853.s002.tif]

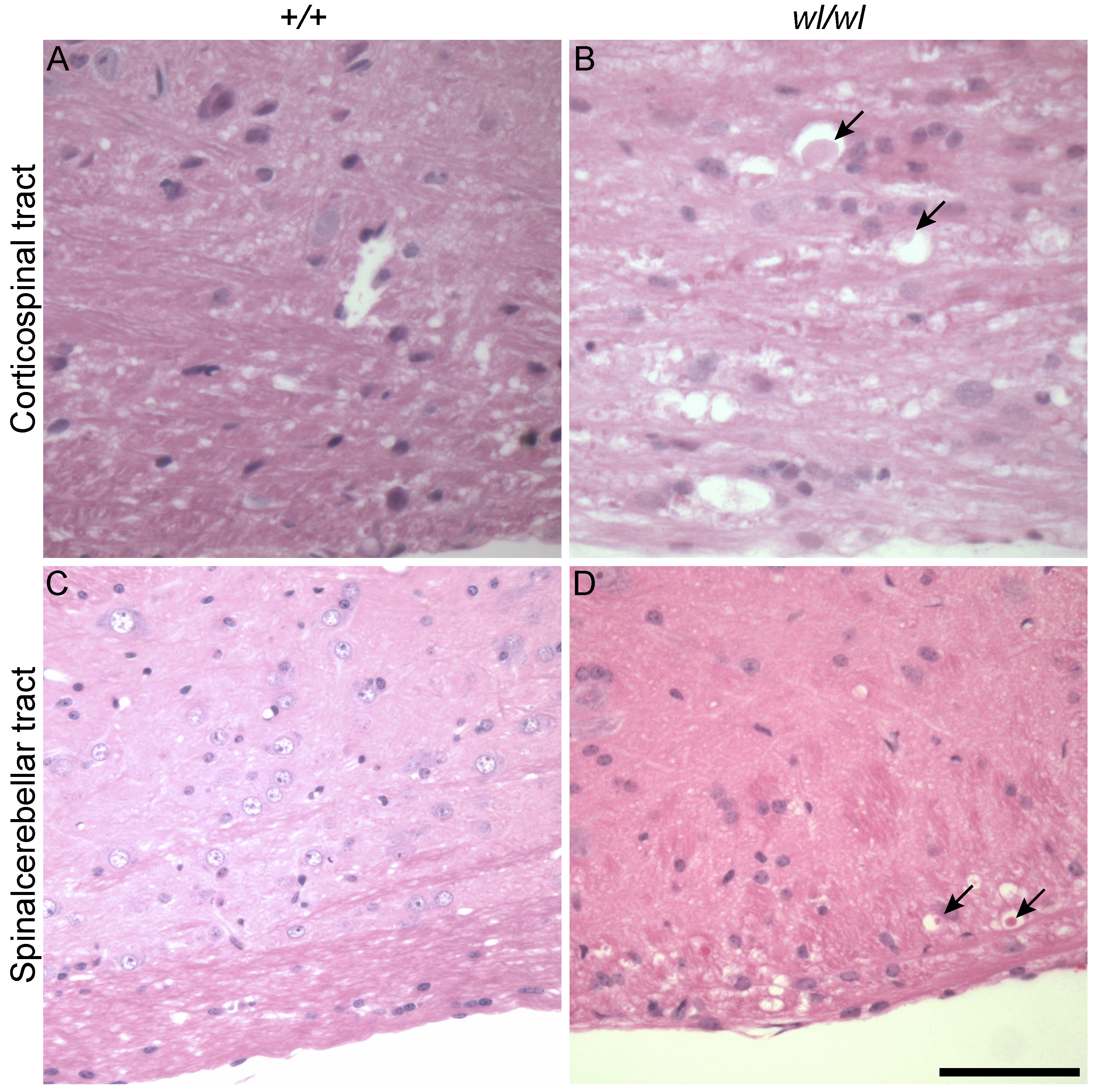

Supplement: Figure S3 — Dystrophic axons in the corticospinal and spinalcerebellar tracts. (A, B) Hematoxylin and eosin staining of the cortocospinal tract in wild type (+/+) and wl/wl mice at two months of age; arrows indicate dystrophic axons. (C, D) Hematoxylin and eosin staining of the spinalcerebellar tract in wild type (+/+) and wl/wl mice; arrows indicate dystrophic axons. Scale bar is 50 µm. (TIF) [file pgen.1002853.s003.tif]

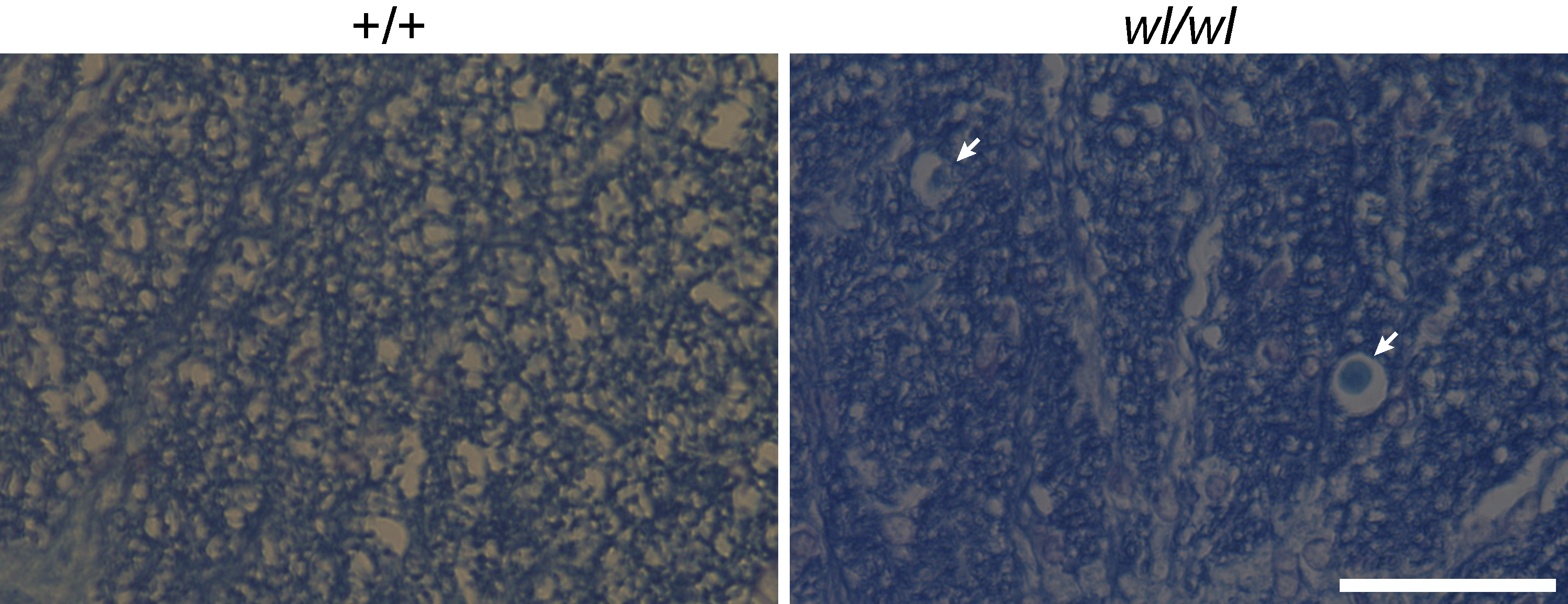

Supplement: Figure S4 — Dystrophic axons in the spinal cord. (A, B) Luxol fast blue staining, another stain that allows for identification of dystrophic axons, of spinal cord sections in wild type (+/+) and wl/wl mice. Arrows indicate dystrophic axons. Scale bar is 50 µm. (TIF) [file pgen.1002853.s004.tif]

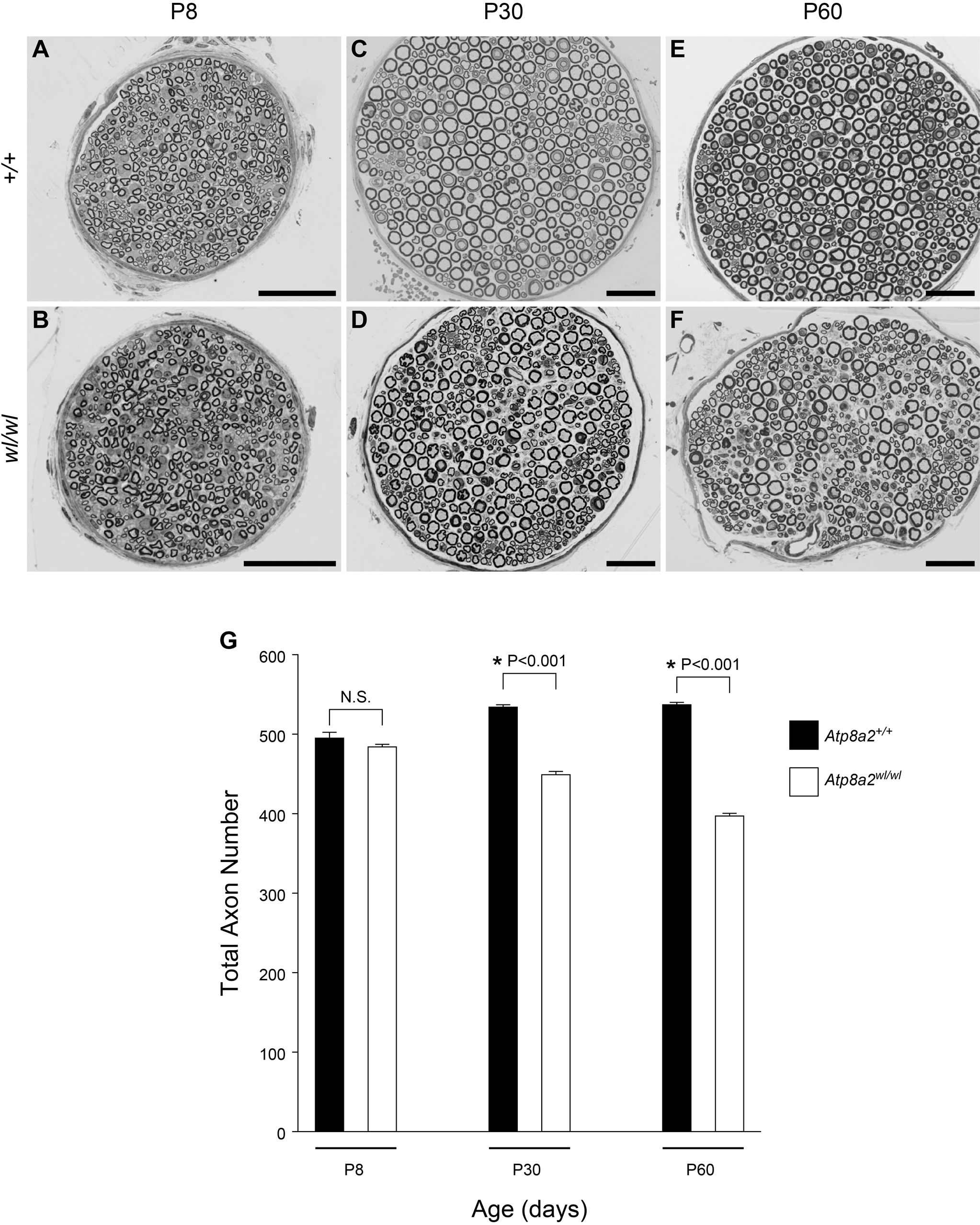

Supplement: Figure S5 — Progressive loss of motor axons in the femoral nerve in wl/wl mice. (A–F) Semi-thin (1 µm) sections of the motor branch of the femoral nerve were stained with toluidine blue and examined for axonal degeneration. There was no obvious difference in the number of axons between mutants and controls at eight days after birth (P8), but there was axons loss in wl/wl nerves at thirty (P30) and sixty (P60) days after birth. (G) Myelinated axons from sections obtained at different ages were counted in control and wl/wl mice. Axons in two sections per mouse for 4 mice of each age and genotype were counted. At P8, no significant change in axon number was found in the motor branch of the nerve in control (495±7) and wl/wl (484±3) mice. However, at P30 a significant decrease in the number of myelinated motor axons was found, with 534±3 axons counted in control mice versus to 449±4 axons counted in wl/wl mice (p<0.01). At P60, significant additional loss of axons was evident in the motor branch with 530±34 axons in wild type control mice versus 397±4 in wl/wl mice (p<0.01). The values are means of axon number in each nerve ± SEM. Scale bar is 50 µm. (TIF) [file pgen.1002853.s005.tif]

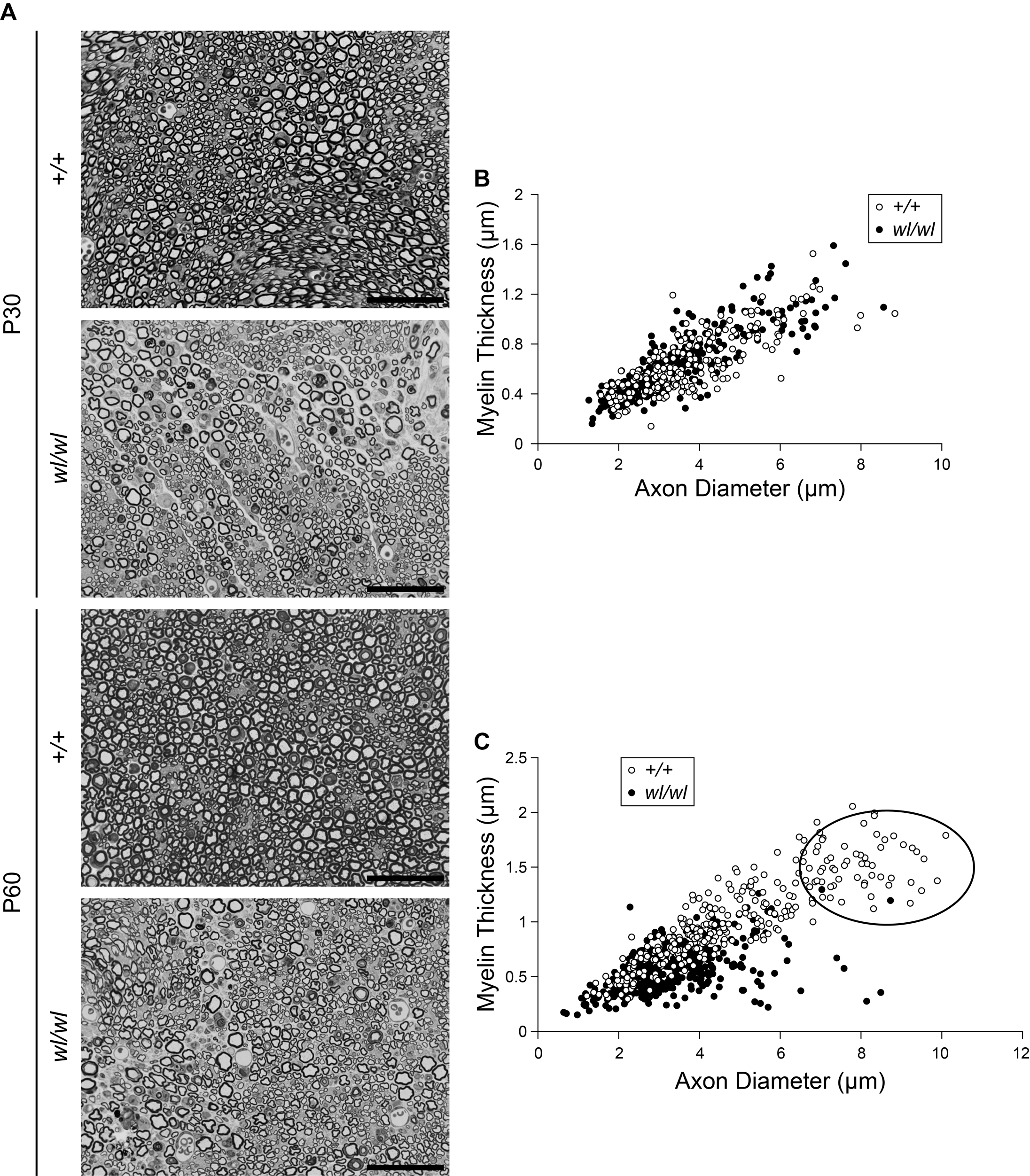

Supplement: Figure S6 — Loss of large diameter axons in the sciatic nerve. (A) Representative semi-thin (1 µm) sections of sciatic nerves obtained from 3 control and 3 wl/wl mice at P30 and P60 were stained with toluidine blue. At P30, sciatic nerves from control (+/+) and mutant (wl/wl) mice were indistinguishable, but at P60 less large diameter axons were present in mutant than in control mice. (B) At P30, the distribution of axonal diameters is similar in control (+/+; with a mean value of 3.45±0.07 µm) and mutant mice (wl/wl; with a mean of 3.62±0.09 µm). A total of 232 axons from three control mice and 333 axons from 4 mutant mice were measured. (C) In contrast, at P60 the mean axonal diameter is decreased from 4.70±0.11 µm in controls to 3.31±0.06 µm in mutants. Consistent with the loss of large-diameter axons, myelin thickness is reduced in mutant mice from a mean of 0.99±0.02 to 0.57±0.01 (p≪0.01, 333 axons from three P60 +/+ mice and 399 axons from four P60 wl/wl mice were analyzed by TEM). Scale bar is 50 µm. (TIF) [file pgen.1002853.s006.tif]

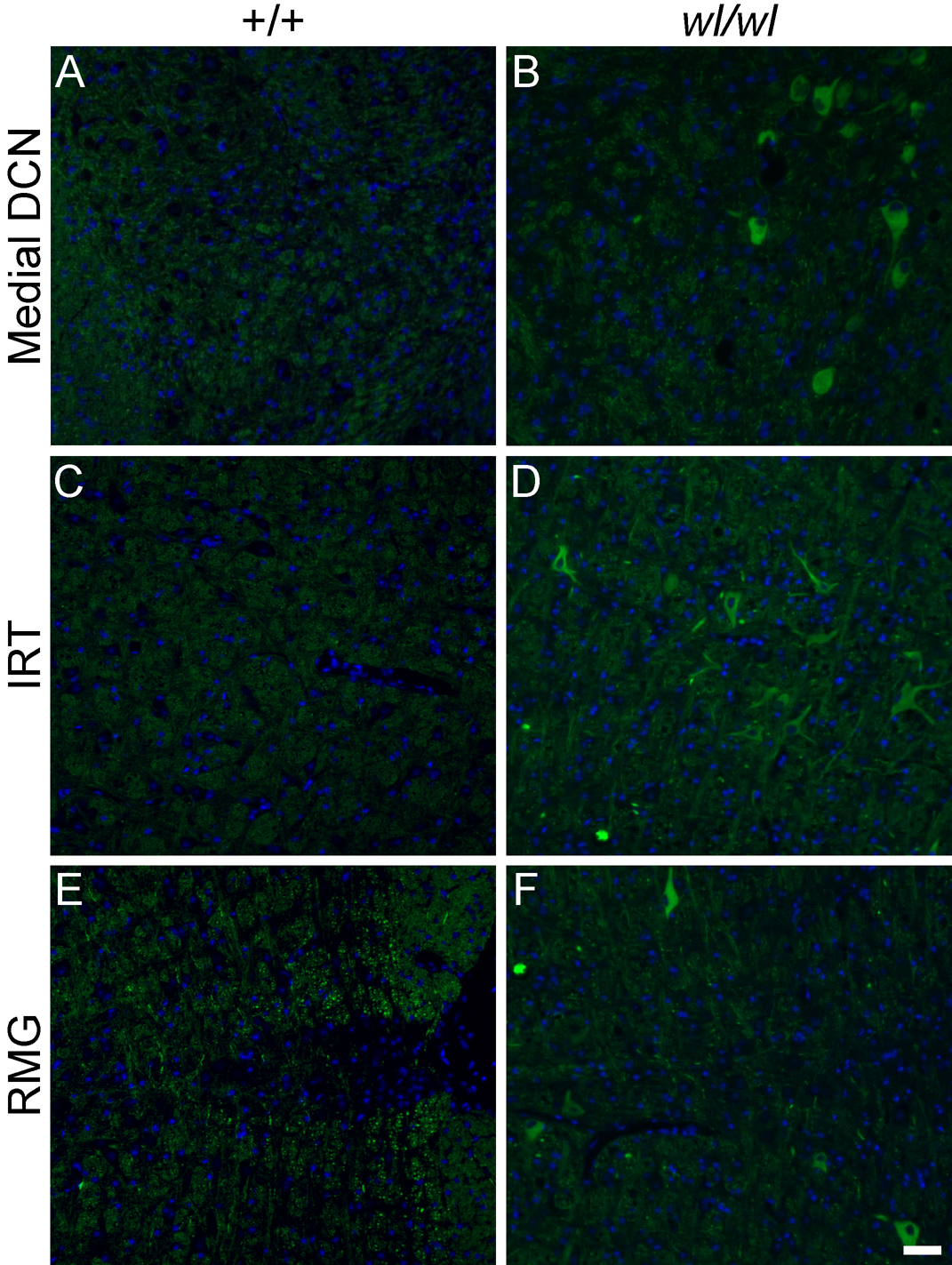

Supplement: Figure S7 — Axon transport defects - Phosphorylated neurofilament (pNF) accumulates in soma of neurons in the cerebellar nucleus, intermediate reticular nucleus and raphe magnus nucleus. (A, B) pNF localized to only the axons in the medial cerebellar nucleus (medial DCN) in wild type mice. It was not present in the soma. In wl/wl mice and indicative of an axon transport defect, however, pNF had accumulated in the somas of neurons of the medial cerebellar nucleus. Similarly, pNF also accumulated in the somas of neurons in the intermediate reticular nucleus (IRT; C, D) and the raphe magnus nucleus (RMG; E, F) of wl/wl mice but not control mice. Tissues were collected from animals at two months of age. Scale bar is 50 µm. (TIF) [file pgen.1002853.s007.tif]

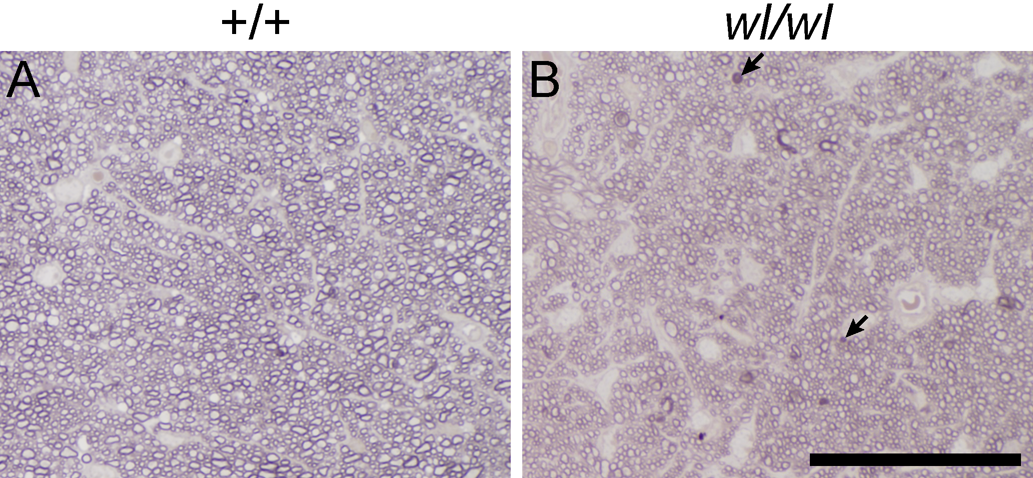

Supplement: Figure S8 — Example of axonal degeneration in the optic nerve detected by PPD staining. (A, B) Representative images of wild type B6 (A) and wl/wl (B) optic nerve semi-thin sections stained with PPD. Optic nerves are from mice at P60, an age where a low level of axonal degeneration is present in wl/wl mice. Arrows indicate damaged axons that stain darkly with PPD. Scale bar is 50 µm. (TIF) [file pgen.1002853.s008.tif]

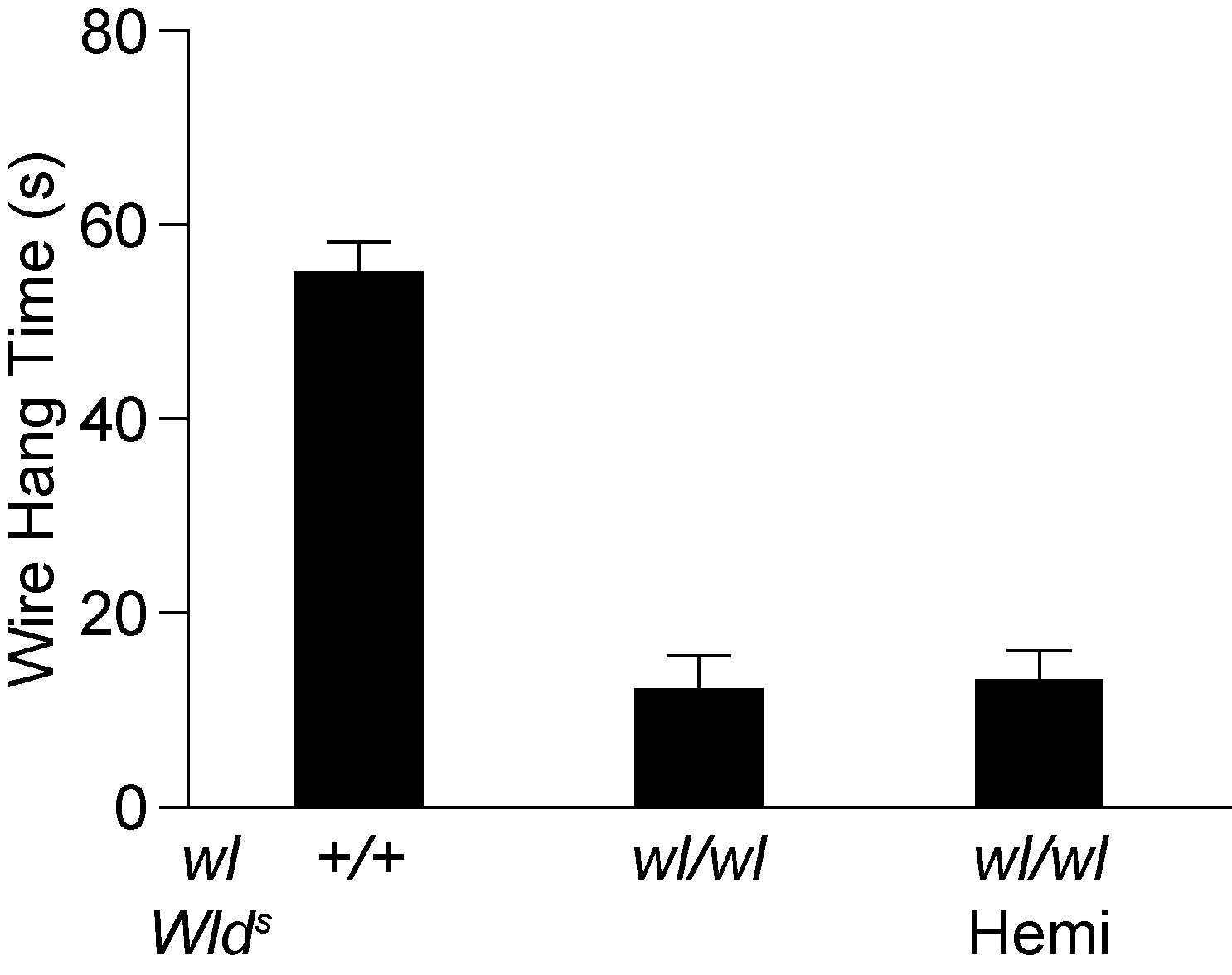

Supplement: Figure S9 — The Wlds gene does not improve performance of wl/wl mice in the wire hang test. In the wire hang test, wl/wl and wl/wl Wlds mice were able to grip the cage top for an average of 12.2±3.4 and 13.2±2.9 seconds respectively (P>0.05), while wild type controls gripped for an average of 55.2±3 seconds. (TIFF) [file pgen.1002853.s009.tiff]

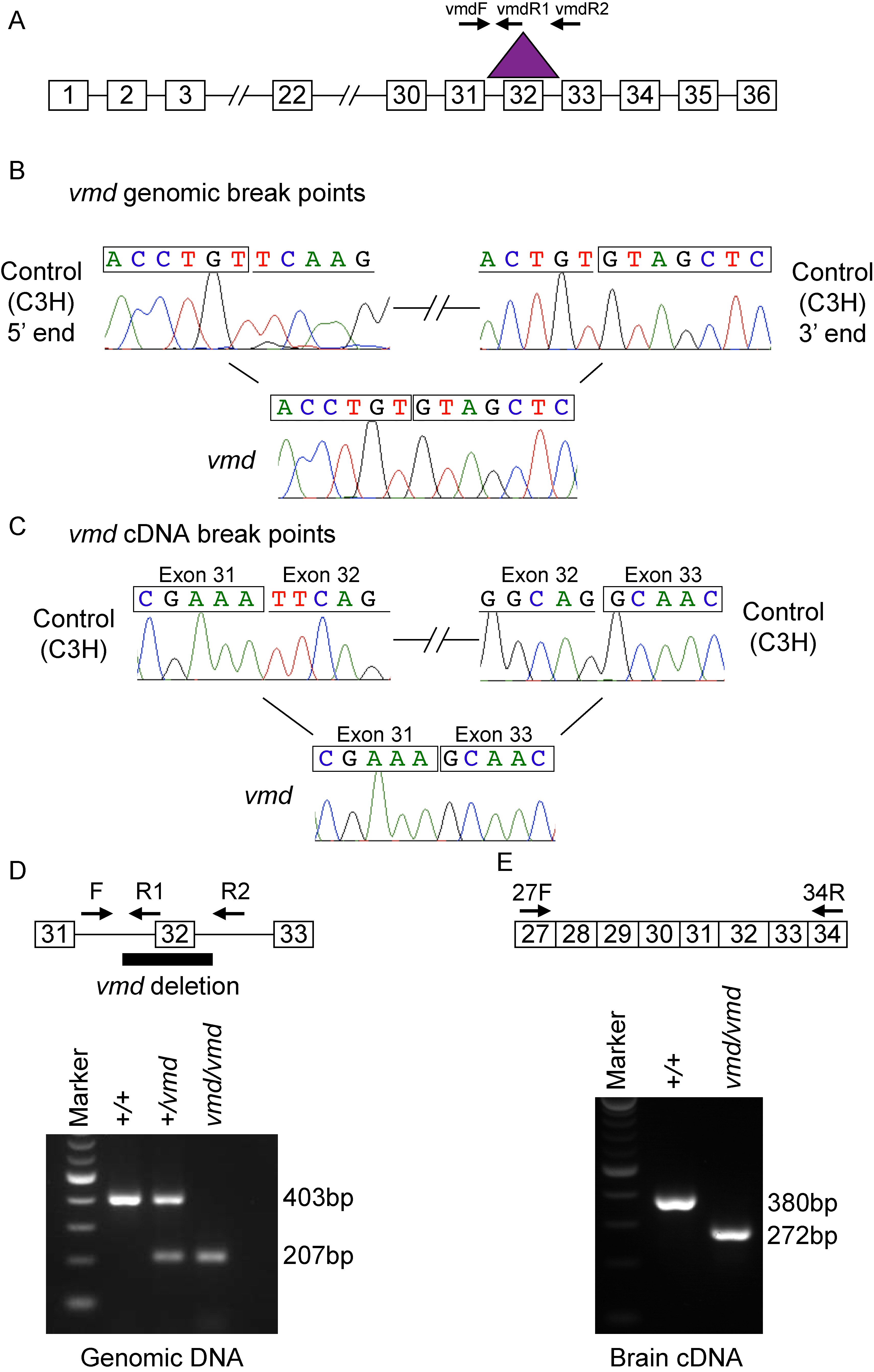

Supplement: Figure S10 — Characterization of the vmd mutation. (A) Diagram showing the structure of the Atp8a2 genomic locus and location of the vmd deletion (indicated by a purple triangle). Primers used for PCR genotyping are indicated by arrows. (B) Sequencing trace showing the deletion in vmd genomic DNA. Deleted sequence is underlined. Nucleotide bases immediately next to the deletion are boxed. vmd 5′ break point: 60,391,594 bp; 3′ break point: 60,382,428 bp. (C) Sequence analysis of vmd cDNA revealed the loss of exon 32 in vmd cDNA. The base pairs deleted by the vmd mutation are underlined, while nucleotide bases immediately flanking the deletion are boxed. (D) PCR based genotyping method used to detect the 9,167 bp deletion in vmd genomic DNA. A band of 403 bp is amplified in control mice using primer F and R1 (+/+). In contrast, a 207 bp product is amplified using primers F and R2 in mutant mice (vmd/vmd). In mice heterozygous for vmd (vmd/+), both products are amplified. (E) Detection of vmd deletion in cDNA by PCR using two primers flanking the deleted region. A product of 272 bp is amplified in vmd cDNA. In contrast, a 380 bp band is observed in control cDNA. (TIF) [file pgen.1002853.s010.tif]

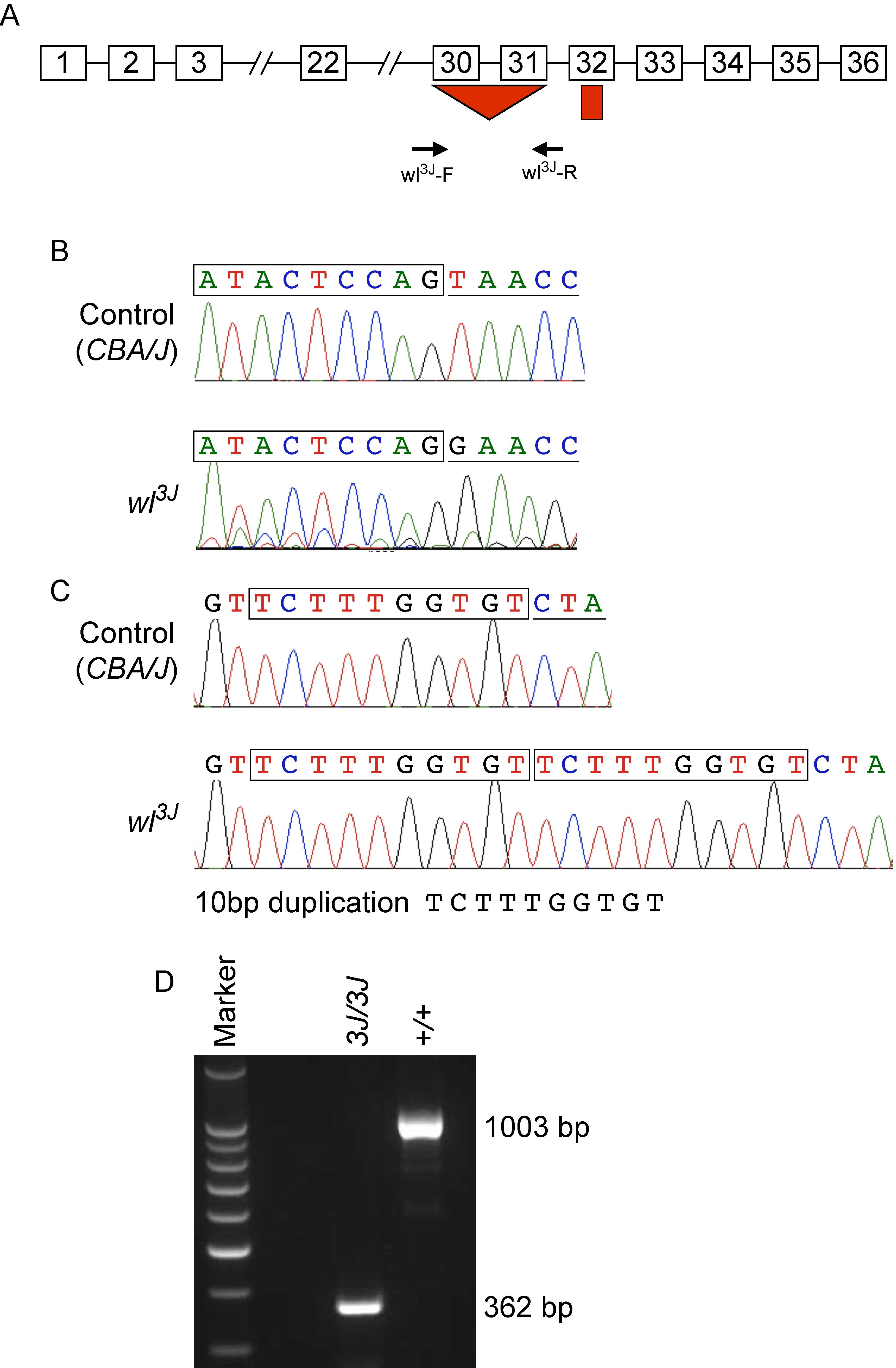

Supplement: Figure S11 — Characterization of the Atp8a2wl3J mutation. (A) Diagram showing the structure of Atp8a2 genomic locus with the 3J mutation indicated using a red triangle (deletion) and rectangle (duplication) respectively. Primers used for PCR genotyping are represented by two arrows. (B) Sequencing trace showing the genomic break points flanking the 3J mutation. The nucleotide bases immediately 5′ to the 641 bp deletion are boxed. (C) The genomic duplication in exon 32 in 3J. The sequence TCTTTGGTGT, which is boxed, is duplicated in 3J DNA. (D) Result of a PCR based genotyping method to detect the 641 bp deletion in 3J genomic DNA using the primers indicated in (A). A band of 1003 bp is amplified in control mice using primer 3J–F and 3J–R. In contrast, a 362 bp product is amplified using the same primer pair with 3J genomic DNA as a template. (TIF) [file pgen.1002853.s011.tif]
